# Computational Modeling Predicts Simultaneous Targeting of Fibroblasts and Epithelial Cells Is Necessary for Treatment of Pulmonary Fibrosis

**DOI:** 10.3389/fphar.2016.00183

**Published:** 2016-06-23

**Authors:** Hayley C. Warsinske, Amanda K. Wheaton, Kevin K. Kim, Jennifer J. Linderman, Bethany B. Moore, Denise E. Kirschner

**Affiliations:** ^1^Department of Microbiology and Immunology, University of Michigan Medical SchoolAnn Arbor, MI, USA; ^2^Department of Internal Medicine, University of Michigan Medical SchoolAnn Arbor, MI, USA; ^3^Department of Chemical Engineering, University of MichiganAnn Arbor, MI, USA

**Keywords:** pulmonary fibrosis, transforming growth factor beta1, fibroblasts, epithelial cells, agent-based modeling, prostaglandin E2, IPF, therapeutics for fibrosis

## Abstract

Pulmonary fibrosis is pathologic remodeling of lung tissue that can result in difficulty breathing, reduced quality of life, and a poor prognosis for patients. Fibrosis occurs as a result of insult to lung tissue, though mechanisms of this response are not well-characterized. The disease is driven in part by dysregulation of fibroblast proliferation and differentiation into myofibroblast cells, as well as pro-fibrotic mediator-driven epithelial cell apoptosis. The most well-characterized pro-fibrotic mediator associated with pulmonary fibrosis is TGF-β1. Excessive synthesis of, and sensitivity to, pro-fibrotic mediators as well as insufficient production of and sensitivity to anti-fibrotic mediators has been credited with enabling fibroblast accumulation. Available treatments neither halt nor reverse lung damage. In this study we have two aims: to identify molecular and cellular scale mechanisms driving fibroblast proliferation and differentiation as well as epithelial cell survival in the context of fibrosis, and to predict therapeutic targets and strategies. We combine *in vitro* studies with a multi-scale hybrid agent-based computational model that describes fibroblasts and epithelial cells in co-culture. Within this model TGF-β1 represents a pro-fibrotic mediator and we include detailed dynamics of TGF-β1 receptor ligand signaling in fibroblasts. PGE_2_ represents an anti-fibrotic mediator. Using uncertainty and sensitivity analysis we identify TGF-β1 synthesis, TGF-β1 activation, and PGE_2_ synthesis among the key mechanisms contributing to fibrotic outcomes. We further demonstrate that intervention strategies combining potential therapeutics targeting both fibroblast regulation and epithelial cell survival can promote healthy tissue repair better than individual strategies. Combinations of existing drugs and compounds may provide significant improvements to the current standard of care for pulmonary fibrosis. Thus, a two-hit therapeutic intervention strategy may prove necessary to halt and reverse disease dynamics.

## Introduction

Pulmonary fibrosis is a pathologic feature associated with many interstitial lung diseases (Buzan and Pop, 2015). A wide range of lung insults can result in development of fibrosis, including antibiotic treatment, infection, and environmental exposures (Vanhee et al., 1994; Daba et al., 2004; Wootton et al., 2011; Zhou et al., 2015). In cases described as idiopathic pulmonary fibrosis (IPF), no explicit cause of fibrosis can be identified (Raghu et al., 2006). Disease presentation includes stiffening and scarring of lungs, decreased flexibility of tissues, and diminished gas exchange (Selman et al., 2004; Swigris et al., 2005; Tzanakis et al., 2005; Maher et al., 2007; Tomioka et al., 2007; Taniguchi et al., 2011). Patients suffering from pulmonary fibrosis have difficulty breathing, reduced quality of life, and ultimately a poor prognosis (De Vries et al., 2001; Jastrzebski et al., 2005; Nishiyama et al., 2005; Swigris et al., 2005; Tzanakis et al., 2005; Tomioka et al., 2007; Zimmermann et al., 2007; Raghu et al., 2010; Verma et al., 2011).

Although mechanisms leading to pulmonary fibrosis are not well-characterized, it is believed that pulmonary fibrosis occurs as the result of dysregulation during the wound healing process (Witte and Barbul, 1997; Diegelmann and Evans, 2004; Strieter, 2008; Hinz et al., 2012). Wound healing occurs in four stages: (I) coagulation and hemostasis, (II) inflammation, (III) proliferation, and (IV) remodeling (Selman et al., 2004). During the third stage of wound healing, fibroblasts proliferate into the wound gap (Midwood et al., 2004). They secrete cytokines, including transforming growth factor-β (TGF-β), which act in both an autocrine and paracrine manner to induce further proliferation and/or eventual differentiation of fibroblasts into myofibroblasts (Desmouliere et al., 1993; Kolodsick et al., 2003; Thannickal et al., 2003; Epa et al., 2015). Myofibroblasts play an important role in the fourth stage of the wound healing process, the remodeling stage. They secrete extracellular matrix (ECM) proteins including collagen and fibronectin (Witte and Barbul, 1997; Midwood et al., 2004; Velnar et al., 2009) that are cross-linked to provide a substrate for re-epithelialization of wounded tissue (Midwood et al., 2004). Myofibroblasts also express α-smooth muscle actin (αSMA), a protein that integrates into actin filaments giving cells a contractile phenotype (Desmouliere et al., 1993; Hinz et al., 2001; Peyton et al., 2008). Through integrin binding, myofibroblasts are able to adhere to surrounding tissue and contract, collapsing the wound gap (Thannickal et al., 2003; Ibrahim et al., 2015). Dysregulation of this process, through unknown mechanisms, results in excessive ECM protein secretion and tissue remodeling. These actions result in the formation of stiff, scarred tissue that is inflexible, and unproductive for gas exchange (Selman et al., 2004; Jastrzebski et al., 2005).

In addition to fibroblasts and myofibroblasts, epithelial cells are a critical component of effective pulmonary wound healing (Camelo et al., 2014). Epithelial cell damage is congruent with pulmonary fibrosis (Adamson and Bowden, 1974; Lama et al., 2002; Maher et al., 2010; Camelo et al., 2014; Prasad et al., 2014). Epithelial cells are an essential component of properly functioning lung tissue. They line the bronchi, airways, and alveoli of the lungs providing a surface for gas exchange and a barrier for infectious agents (Mayer and Dalpke, 2007; Crystal et al., 2008; Holtzman et al., 2014). During pulmonary injury, epithelial cells are damaged and repair is needed in order to restore functionality to the wounded tissue (Adamson, 1984; Prasad et al., 2014; Epa et al., 2015). During fibrosis, excessive secretion of pro-fibrotic cytokines such as TGF-β1 produces an environment that is toxic to epithelial cells (Willis and Borok, 2007; Crosby and Waters, 2010). Furthermore, excessive tissue remodeling can induce further epithelial cell damage, loss of epithelial protective factors and tissue contraction reducing the surface area available for re-epithelialization (Krieg et al., 2007; Hinz et al., 2012).

**Figure 1:**
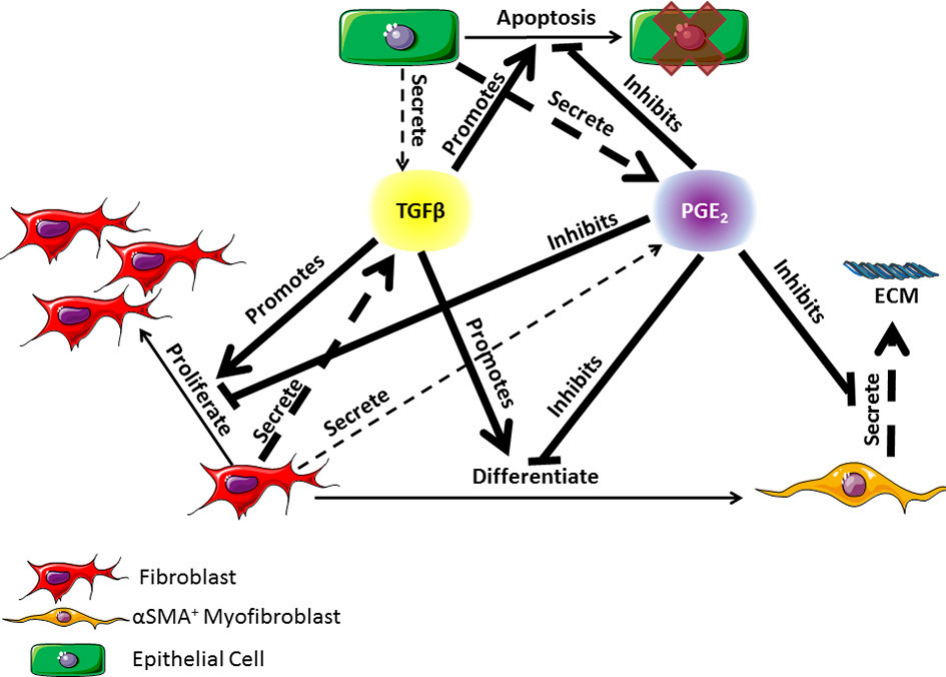
**Diagram of the co-regulatory relationship between fibroblasts, myofibroblasts, and epithelial cells through TGF-β1 and PGE_**2**_ signaling occurring in lung tissue**. TGF-β1 is primarily secreted by fibroblasts but can also be secreted in small part by epithelial cells (Willis and Borok, 2007). PGE_2_ is primarily secreted by epithelial cells but can also be secreted in small part by fibroblasts (Lama et al., 2002; Moore et al., 2003). TGF-β1 can promote fibroblast proliferation or differentiation into α-smooth muscle actin positive myofibroblasts, and epithelial cell apoptosis (Desmouliere et al., 1993; Kolodsick et al., 2003; Thannickal et al., 2003; Epa et al., 2015). PGE_2_ can inhibit the actions of TGF-β1 and can also inhibit myofibroblast secretion of extracellular matrix (ECM) proteins (Fine et al., 1989; Moore et al., 2003; Thannickal et al., 2003; Thomas et al., 2007; Tian and Schiemann, 2010; Epa et al., 2015). Dashed arrows indicate secretion of a molecule. The thickness of the arrow indicates relative contribution of the cell type to the mediator concentration. Solid lines indicate an action of the cytokine on a given cell type. Arrows indicate a positive effect on the cell while bar headed lines indicate a negative effect. ECM is the extracellular matrix.

At the apex of cell-cell interactions during pulmonary wound healing are two key classes of cytokines: pro-fibrotic mediators and anti-fibrotic mediators. TGF-β1, the most well characterized pro-fibrotic mediator in pulmonary fibrosis, is a cytokine secreted by a wide range of cell types including fibroblasts, with effects that are cell type and tissue specific (Coffey et al., 1988; Pietenpol et al., 1990; Bendelac et al., 1997; Thannickal et al., 2003; Kronenberg and Rudensky, 2005; Leveen et al., 2005; Marie et al., 2006; Li et al., 2007; Liu et al., 2008). TGF-β1 is an autocrine and paracrine signal that can induce fibroblast proliferation and/or differentiation into myofibroblasts (Figure 1) (Lehnert and Akhurst, 1988; Turley et al., 1996; Tobin et al., 2002). Growth factors are necessary for fibroblast proliferation and TGF-β1 is representative of these growth factors. TGF-β1 is also able to induce fibroblast to myofibroblast differentiation, which can be measured by the presence of αSMA in cultured cells (Figure 1) (Fine and Goldstein, 1987; Desmouliere et al., 1993; Thannickal et al., 2003). High concentrations of TGF-β1 have been shown to induce alveolar epithelial cell apoptosis (Figure 1) (Yanagisawa et al., 1998; Hagimoto et al., 2002; Kuwano, 2008) and are therefore detrimental to epithelial cell survival during wound healing. In opposition to TGF-β1, PGE_2_ is a well characterized anti-fibrotic lipid mediator with a wide range of influence that is cell and tissue type specific. In pulmonary tissues, PGE_2_ is predominantly secreted by epithelial cells as an inducer of fibroblast quiescence (Lama et al., 2002; Moore et al., 2003). It also serves as a negative regulator of TGF-β1, inhibiting fibroblast proliferation and differentiation, myofibroblast ECM secretion, and TGF-β1 induced epithelial cell apoptosis (Figure 1) (Fine et al., 1989; Kolodsick et al., 2003; Moore et al., 2003; Tian and Schiemann, 2010; Epa et al., 2015). We consider that PGE_2_ is representative of mediators that inhibit fibroblast activation. Epithelial cells, fibroblasts, and myofibroblasts all play important roles in lung function and pulmonary wound healing (Diegelmann and Evans, 2004; Strieter, 2008; Crosby and Waters, 2010; Guo and Dipietro, 2010; Camelo et al., 2014). Their interactions and co-regulation are paramount to understanding the mechanisms underlying dysregulation of the wound healing process and the development of fibrosis (Figure 1). Recent work by Epa et al. (2015) shows that alveolar epithelial cells from healthy lungs are able to inhibit TGF-β1-induced fibroblast differentiation and secretion of ECM proteins in a PGE_2_ dependent manner *in vitro* (Epa et al., 2015). Recent systems biology and modeling approaches by our group further demonstrate the importance of PGE_2_ in regulating the activation of fibroblasts (Warsinske et al., 2015). As observed in other systems, it is likely that a balance of both positive and negative regulators (e.g., TGF-β1 and PGE_2_ respectively) is necessary for achieving homeostasis and avoiding excessive fibroblast activation (Cilfone et al., 2013; Warsinske et al., 2015). PGE_2_ is also shown to protect epithelial cells from toxicity of pro-fibrotic mediators like TGF-β1 (Saha et al., 1999). Together TGF-β1 and PGE_2_ serve as examples of positive and negative regulators to preserve balance in the responses of epithelial cells, fibroblasts, and myofibroblasts to tissue damage (Figure 1).

Treatments for pulmonary fibrosis are limited. Lung transplantation was considered the only available intervention until recently. In October of 2015, two drugs, Nintedanib and Pirfenidone, were approved by the United States Food and Drug Administration (FDA) for the treatment of IPF (George et al., 2016). Neither of these available therapies is curative. Both treatments slowed but did not halt or reverse the progress of IPF marked by a reduction in the decline of patients forced vital capacity (FVC) (King et al., 2014; Kreuter, 2014; Lederer et al., 2015; Richeldi et al., 2015; Costabel et al., 2016).

Both drugs target the dynamics of fibroblasts, namely inhibiting proliferation, differentiation, and TGF-β1 production. However, neither nintedanib nor pirfenidone have been demonstrated to promote the survival or regeneration of epithelial cells in a fibrotic lung. There is evidence that pirfenidone may even inhibit retinal epithelial cells (Wang et al., 2013).

Here we construct an *in silico* model that captures the co-regulation of fibroblasts and epithelial cells *in vitro.* There is substantial support for constructing agent-based models (ABMs) *of in vitro* co-culture systems. These models are used to study a wide range of processes including, but not limited to wound healing (Maini et al., 2004; Walker et al., 2004; Mi et al., 2007; Stern et al., 2012), tissue patterning (Thorne et al., 2007), and tumor progression (Mansury et al., 2002; An et al., 2009; Zhang et al., 2009). The construction of this model is based on previous work in our lab building a 3D model of granuloma formation in the lung.

With this model, we seek to identify which mechanisms of co-regulation determine fibroblast and epithelial cell outcomes during wound healing. By capturing a wide range of possible outcomes we are able to predict which mechanisms would be good potential therapeutic targets for preventing and reversing fibrosis. We hypothesize that a two-hit approach targeting specific mechanisms to both inhibit fibroblast dysregulation and simultaneously promote epithelial cell survival is necessary to halt or reverse damage associated with pulmonary fibrosis. In order to construct the model and test this hypothesis, we take a systems biology approach that combines *in vitro* experiments with *in silico* simulations of a co-culture system. We use a multi-scale, hybrid ABM to identify mechanisms that simultaneously and independently drive fibroblast dysregulation and epithelial cell death. We take a reductionist approach to fibrosis, looking solely at the cells that are experiencing damage (epithelial cells) or inflicting damage (fibroblasts and myofibroblasts) and model representative pro- and anti-fibrotic mediators rather than a more complex tissue environment. This co-culture environment enables us to focus our search for mechanisms driving outcomes for these cells while limiting potentially confounding factors. It also allows us to look specifically at co-regulation of fibroblasts and epithelial cells and concurrently compare the effects of intervention strategies. Our model enables us to predict whether a two-hit synergistic therapeutic strategy for pulmonary fibrosis addressing multiple aspects of the disease will be the most successful at improving cellular outcomes.

## Methods

### *in vitro* studies of fibroblast proliferation

The IMR-90 normal human lung fibroblast cell line was obtained from American Type Culture Collection (ATCC; CCL-186). Approximately 5000 cells/well are plated onto each well of a 96 well plate and either left untreated, treated with 0.1–4 ng/ml of activated TGF-β1, or treated with 0.1–100 nM of PGE_2_. Cells were treated with 10 μCi of radioactive thymidine (Fisher) at 32 h and harvested at 48 h post treatment. Cells were harvested onto glass fiber filters using an automated cell harvester and filters were counted using a beta scintillation counter. Proliferation was compared by counts per minute (cpm) between samples using ANOVA with a *post-hoc* Sidak multiple comparisons test.

### *in vitro* studies of fibroblast differentiation

Approximately 75,000 IMR-90 cells were plated into 8-well Titer-tek slides and cultured in SFM for 16 h to synchronize cells and restore basal levels of αSMA. After 16 h the cells were left untreated, or treated with 0.1, 0.5, 1.0, 2.0, or 4.0 ng/ml of TGF-β1 for 24 h. Cells were then blocked with 1% FBS and stained with a 1:500 fold dilution of anti-αSMA (Sigma F3777) fluorescently-conjugated antibody. The cell nuclei were stained with DAPI and coverslips were added using Vectashield (Vector brand) H1200. Three to five fields per well were counted (at least 300 total cells) and the proportion of total cells expressing αSMA was determined.

### *in vitro* studies of epithelial cell death

Type II alveolar epithelial cells were isolated from wild type mice per standard protocol (Corti et al., 1996) and then plated on Matrigel (BD Biosciences) in Small Airways Growth Media (SAGM) (Lonza) with 5% FBS (Hyclone) and 10 ng/ml KGF (PeproTech). Twenty-four hours later cells were trypsinized and replated in serum free SAGM on Matrigel in a 24-well plate. Seventy thousand cells were plated in each well. Twenty-four hours later, the cells were treated with TGF-β1 added at different concentrations as well as IncuCyte Caspase-3/7 Reagent for Apoptosis (Essen BioScience) at 5 μM, according to the manufacturer's instructions. This reagent is cleaved by activated caspase-3/7 resulting in nuclear fluorescent staining. Twenty-four hours later, cells were analyzed under a fluorescent microscope. The percent of fluorescent cells was manually counted for each dose of TGF-β1. We had four replicate wells and counted 2–4 fields per each replicate a total of 8–16 fields for each dose of TGF-β1.

### Multi-scale model construction

#### Cellular scale agent-based model

**Figure 2:**
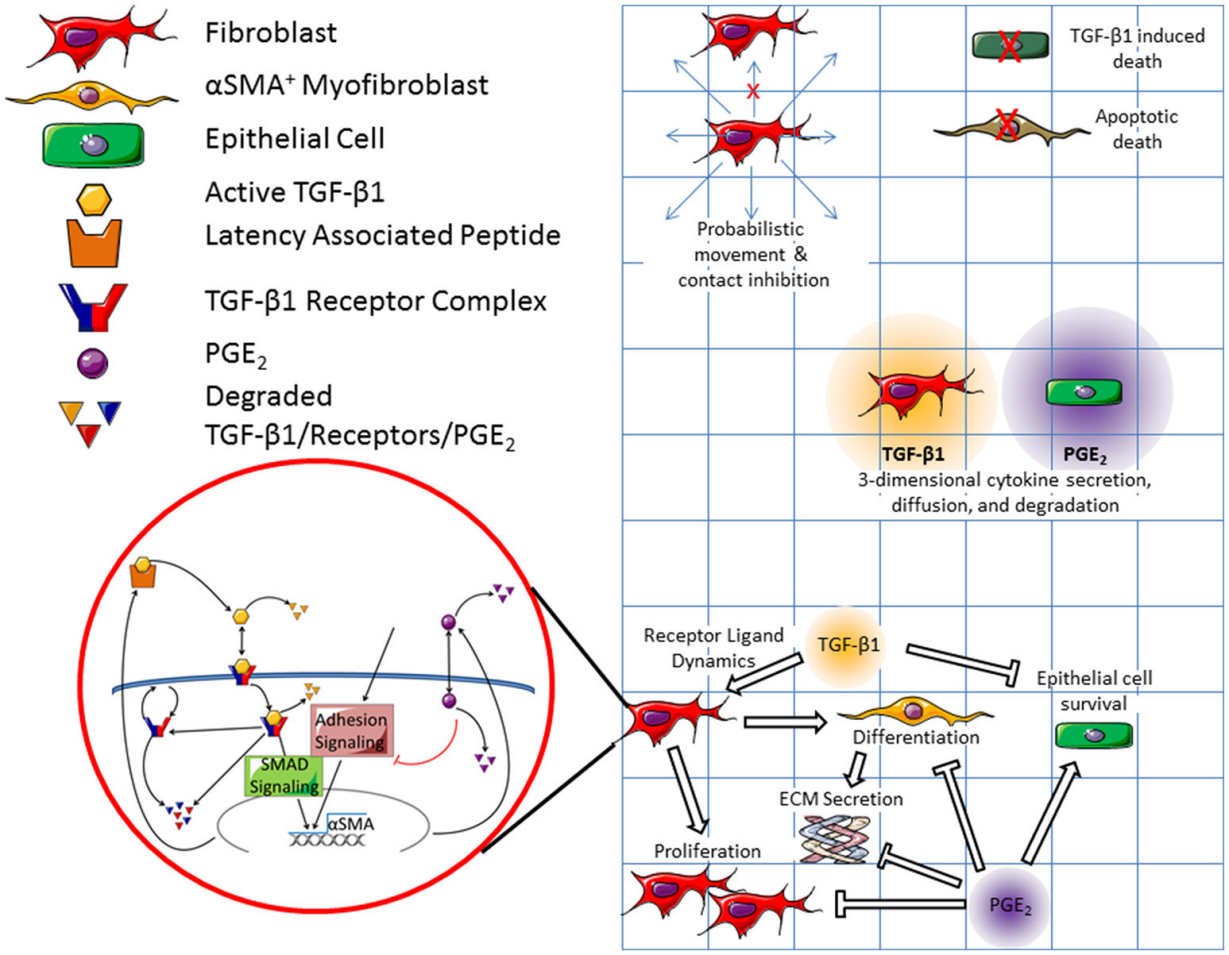
**Schematic representation of both cellular and molecular scale physiological interactions in the hybrid multi-scale computational co-culture model showing linking of models**. The cellular scale model (represented by the grid) contains three cell types; fibroblasts, myofibroblasts, and epithelial cells. At the cellular scale fibroblasts are capable of probabilistic movement which is inhibited by the presence of other cells (only a single cell can occupy a grid space at a given time). Epithelial cells and myofibroblasts can undergo TGF-β1 induced death and apoptotic death respectively. Fibroblasts secrete TGF-β1, which diffuses in 3D. Epithelial cells secrete PGE_2_, which also diffuses in 3D. All cell types respond to chemical concentrations present in their local environment. We coarse-grained epithelial-cell binding dynamics for both TGF-β1 and PGE_2_. Fibroblasts and myofibroblasts have coarse-grained binding dynamics for PGE_2_, and fine grained-binding dynamics of TGF-β1. The fine grained TGF-β1 binding dynamics are defined by the molecular scale mathematical model that runs within each fibroblast and myofibroblast agent (shown in red bubble). Within this model active TGF-β1 (yellow hexagon) is bound by the TGFβ1 receptor complex (blue and red) on the surface of the cell. Bound receptors are internalized and can induce downstream SMAD signaling ultimately inducing the synthesis of αSMA and latent TGF-β1 (yellow and orange polygon). Following signaling, the internalized TGF-β1 is degraded and the receptor complex is either degraded or restored to the surface of the cell. PGE_2_ (purple circle) is up-taken by cells and serves as a negative regulator of αSMA and TGF-β1 synthesis. Arrows indicate effects that promote behaviors, while bar headed lines indicate inhibitory effects. Contents within the red circle depict a previously published molecular scale model of TGF-β1 signaling (Warsinske et al., 2015) that is operational within all fibroblasts in the model.

ABMs are a class of *in silico* models that demonstrate how system level dynamics can emerge as a result of the adaptive behavior of individuals. They do this by assigning probabilistic actions and traits to individual agents within a system, and then tracking these actions over time and space. These models have a defined domain where agents can interact with each other and their environment. Agents (cells) are tracked in a discrete fashion (i.e., counted) and therefore some ABMs can provide spatial outputs as well as numerical outputs. Because the behaviors of agents can be probabilistic, ABMs are stochastic in nature. Our ABM consists of three cellular agent types: fibroblasts, myofibroblasts, and epithelial cells. The environment of our ABM simulates an *in vitro* cell culture plate representing a cubic area of 1.73 × 1.73 × 6.20 mm, or about one tenth the volume of a well in a 96 well plate (Figure 2). We choose to model one tenth of the well to reduce the computational burden of the three dimensional (3D) model. Our simulated plate is partitioned into a grid with side lengths of 81 compartments (81 × 81) and makes up the first layer of our model. There are 6561 compartments in each layer of the model and 282 layers in total. The bottom layer represents the surface of the dish. The 281 layers above the dish make up a large media compartment that represents the depth of a plate (~6.2 mm). There are a total of 1,850,202 compartments in the model with a total volume of ~19.7 μL. The construction of this model is based on our previous work with ABMs, including unpublished work with 3D models (Segovia-Juarez et al., 2004; Ray et al., 2009; Fallahi-Sichani et al., 2011; Cilfone et al., 2013, 2015a; Chang et al., 2015).

Within this framework, agents follow cell-type-specific rules capturing physiological interactions tracked at the cellular scale (Figure 2). Cellular interactions are guided by rules that include movement of fibroblasts on the surface of the plate, cellular contact inhibition; myofibroblast cell death, TGF-β1 mediated epithelial cell death, TGF-β1- and PGE_2_-mediated fibroblast proliferation, fibroblast differentiation into myofibroblasts, and myofibroblast secretion of ECM. For a full list of cellular model rules, see Supplementary Material. Cells in our simulated culture dish, like cells *in vitro*, are adhesion-dependent, meaning that they adhere to the surface of the plate (Thannickal et al., 2003). ECM and latent TGF-β1 also adhere to the bottom of the plate (Shi et al., 2010). The ECM proteins are assumed to be cross-linked, forming a matrix on the bottom of the plate (Shi et al., 2010). Latent TGF-β1 adheres to this matrix. When TGF-β1 is activated, the active molecule is released from the matrix and can diffuse through the media.

#### Molecular scale ordinary differential equation model

Figure 2 shows a schematic of the molecular scale model. We use our ordinary differential equation (ODE) model of fibroblast receptor-ligand dynamics described previously (Warsinske et al., 2015). Briefly, non-linear ODEs capture not only TGF-β1 synthesis, degradation, activation, receptor binding, and dissociation but also receptor dynamics including synthesis, internalization, recycling, and degradation of the TGF-β1 receptor complex. The model provides coarse-grained dynamics for SMAD and Rho/ROCK signaling as well as for adhesion and PGE_2_ signaling in fibroblasts. This earlier work identified the need for both a positive and negative regulator to achieve homeostatic fibroblast activation. For example, periodic signaling from either TGF-β1 (an example positive regulator) or PGE_2_ (an example negative regulator) was insufficient to produce controlled fibroblast activation.

Active TGF-β1 and PGE_2_ are secreted from cells or released from the ECM into the media compartment where they diffuse in 3D (Cilfone et al., 2015b). Both TGF-β1 and PGE_2_ can degrade over time. Extracellular mediator concentrations influence αSMA synthesis by fibroblasts and myofibroblasts, as detailed in the molecular model described below. Similarly, αSMA from the molecular model described below drives fibroblast differentiation (see list of all rules in Supplementary Material).

#### Linking molecular and cellular scale models

We capture both molecular and cellular actions in a multi-scale hybrid model by linking the cellular scale virtual co-culture ABM and molecular scale TGF-β1dynamics ODE model described above using techniques previously described (Fallahi-Sichani et al., 2011; Cilfone et al., 2013, 2015a,b). In particular, an output from the molecular level model—αSMA—is a feed forward input into the cellular level model, driving fibroblast to myofibroblast differentiation. The concentration of TGF-β1 and PGE_2_, secreted by fibroblasts and epithelial cells, are outputs of the cellular scale model and inputs into the molecular scale model of fibroblast activation. These mediators dictate αSMA synthesis in the molecular scale model completing the connection between the two models and scales. Thus, the cellular and molecular scale models are connected by extracellular levels of TGF-β1 and PGE_2_, and intracellular αSMA (Figure 2).

### Parameter derivation and estimation

Parameter values are identified from published experimental work, estimated from experiments herein, and/or predicted using uncertainty analysis. The rate constants for latent and active TGF-β1 degradation were derived from half-lives published by Wakefield et al. (9.2 ± 1.4 min and 2.7 ± 0.4 min respectively) assuming first order kinetics (Wakefield et al., 1990). Additional parameters obtained from the literature are as described in Warsinske et al. (2015).

**Figure 3:**
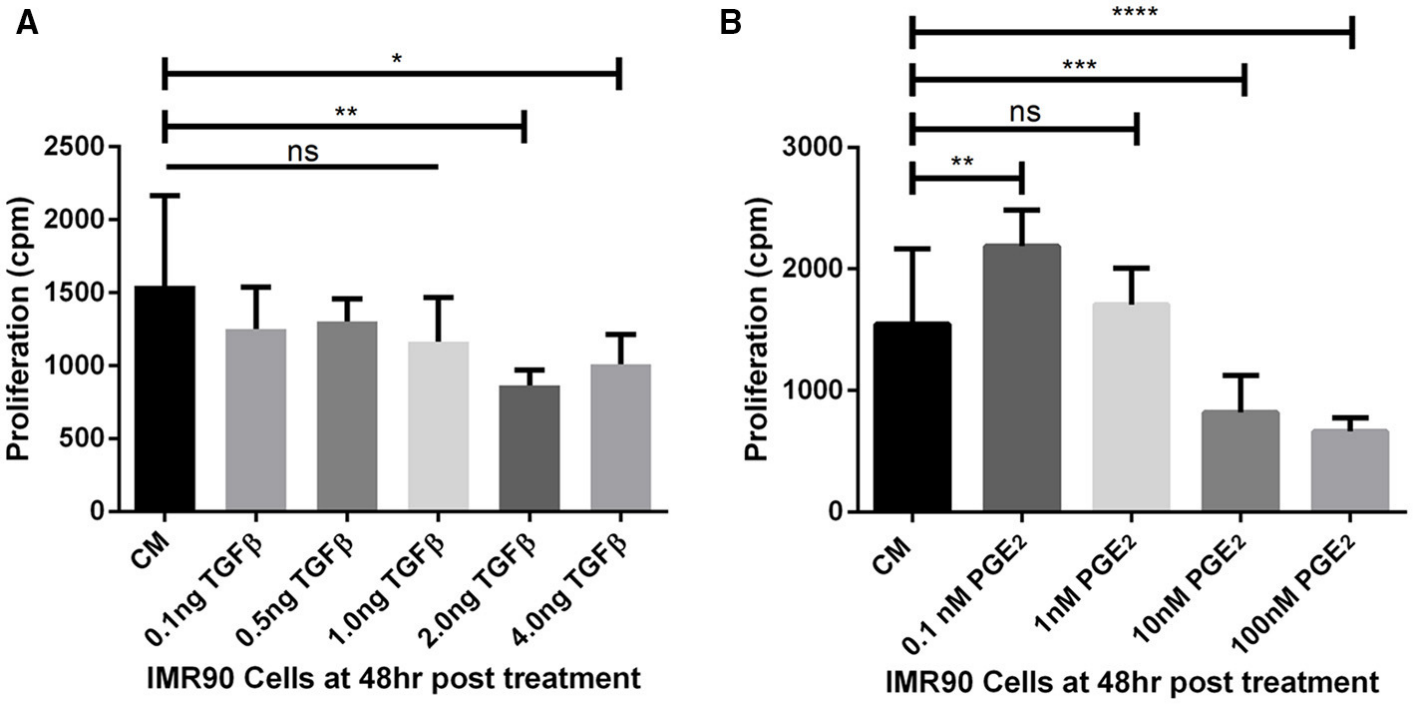
**Estimating fibroblast proliferation thresholds modulated by TGF-β1 and PGE_**2**_**. **(A)** IMR90 fibroblasts were cultured in complete media (CM) and treated with 0.1, 0.5, 1.0, 2.0, or 4.0 ng/ml of TGF-β1 for 48 h in the presence of radioactive thymidine. Proliferation was measured in counts per minute (cpm). Sidak's multiple comparison test was used to determine significance (Salkind and Abdi, 2007). Fibroblast proliferation was significantly reduced compared to CM alone in the presence of 2.0 and 4.0 ng/ml of TGF-β1. **(B)** IMR90 fibroblasts were cultured in CM alone or with 0.1, 1.0, 10.0, 100.0 nM of PGE_2_ for 48 h in the presence of radioactive thymidine. Proliferation was measured in cpm. Sidak's multiple comparison test was used to determine significance (Salkind and Abdi, 2007). Fibroblast proliferation was significantly increased compared to CM alone in 0.1 nM PGE_2_ and significantly decreased in 10 and 100 nM PGE_2_. There was no difference in proliferation between fibroblasts cultured in CM and 1 nM PGE_2_. The significance line without the end irons indicates *p* > 0.05 for all comparisons under the line. ^*^p < 0.05 ^**^p < 0.01 ^***^p < 0.001 ^****^p < 0.0001.

The threshold concentrations of TGF-β1 and PGE_2_ that allow or inhibit fibroblast proliferation *in vitro* were derived from experiments herein. Fibroblasts proliferate in concentrations of TGF-β1 up to 1 ng/ml as compared to fibroblasts cultured in complete media (CM) alone (Figure 3A). At 2 and 4 ng/ml fibroblast proliferation is significantly reduced compared to untreated cells, likely due to myofibroblast differentiation. Fibroblasts proliferate uninhibited in concentrations of PGE_2_ up to 1 nM (Figure 3B). At 0.1 nM PGE_2_ fibroblasts proliferate significantly more than fibroblasts cultured in CM alone (Figure 3B). Fibroblast proliferation is significantly inhibited at 10 and 100 nM concentrations of PGE_2_ (Figure 3B). Fibroblasts cultured in complete media had a doubling time of ~24 h (data not shown).

Parameters estimated using uncertainty analysis (described below) include soluble PGE_2_ degradation rate constant, fibroblast sensitivity to PGE_2_, probability of fibroblast movement, αSMA synthesis rate, max αSMA, and level of TGF-β1 lethal to epithelial cells.

### Cellular-scale model calibration

**Figure 4:**
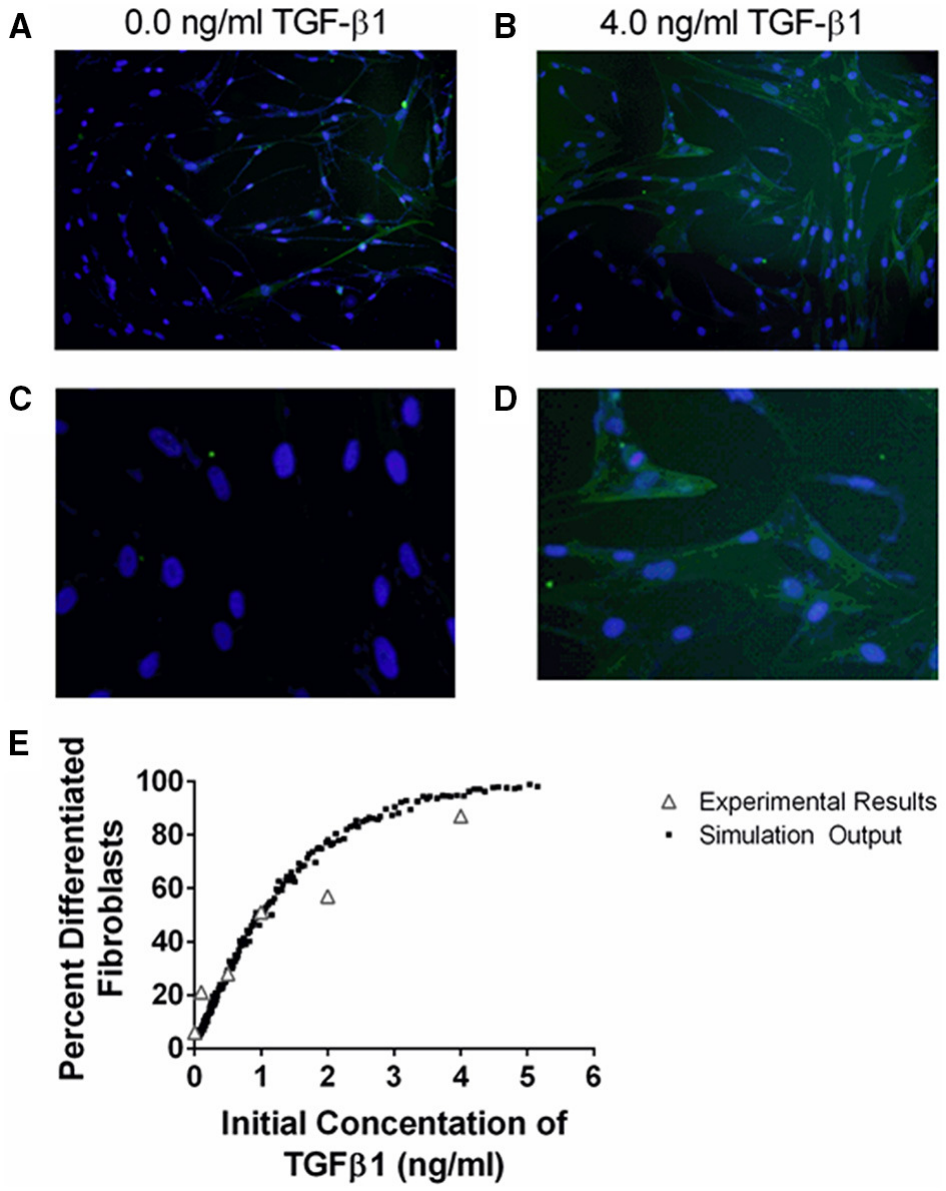
**Comparison between data and computational model fit for fibroblast differentiation at 24 h in response to TGF-β1 treatment**. IMR90 fibroblasts were cultured in serum free media (SFM) alone or with an additional 0.1, 0.5, 1.0, 2.0, 4.0 ng/ml acid activated TGF-β1. The proportion of differentiated cells at 24 h was determined by staining with anti-αSMA and DAPI. **(A)** Representative image of fibroblasts cultured in SFM alone at 100x magnification. **(B)** Representative image of fibroblasts cultured in 4.0 ng/ml TGF-β1 at 100x magnification. **(C)** Representative images of fibroblasts cultured in SFM alone at 400x magnification. **(D)** Representative images of fibroblasts cultured in 4.0 ng/ml TGF-β1 at 400x magnification. **(E)** Open gray triangles indicate experimental results. Solid black squares indicate simulation output. The proportion of either experimental or simulated fibroblasts having undergone differentiation at 24 h is compared to the initial concentration of active TGF-β1 in the tissue culture or simulation. The simulation data closely matches the experimental data.

**Figure 5:**
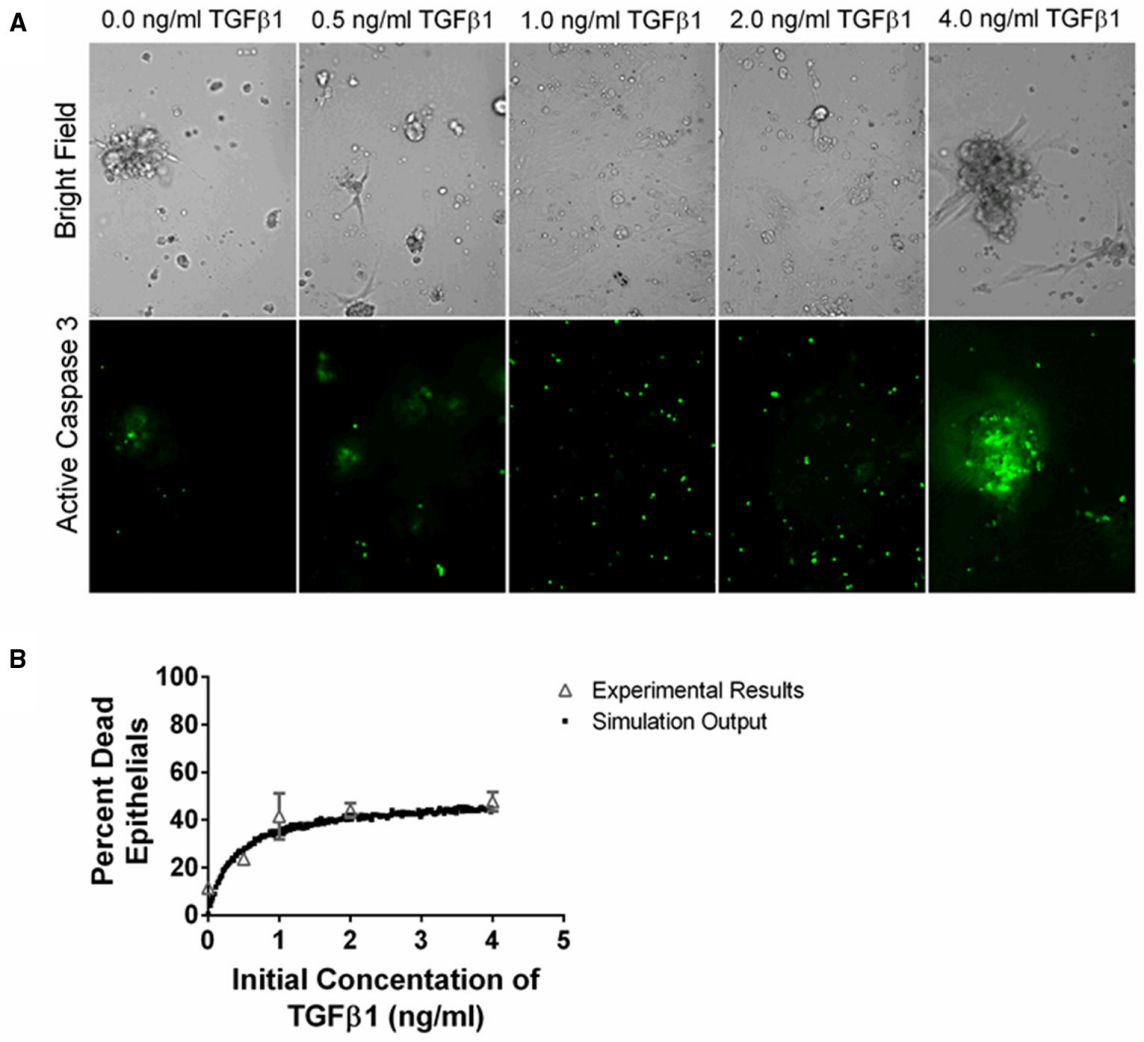
**Comparison between data and computational model fit for epithelial cell survival at 24 h in response to TGF-β1 treatment**. Type II alveolar epithelial cells were cultured in SFM alone or with an additional 0.5, 1.0, 2.0, 4.0 ng/ml acid activated TGF-β1. The number of caspase 3 positive cells was evaluated at 24 h by fluorescent staining. **(A)** Representative image of cell cultures treated with 0.0, 0.5, 1.0, 2.0, or 4.0 ng/ml of TGF-β1 at 200x magnification. These images show an increase in caspase 3 activation of the epithelial cells in the presence of increasing concentrations of TGF-β1. **(B)** Open gray triangles indicate experimental results. Solid black squares indicate simulation output. We compare the proportion of experimental or simulated epithelial cells having undergone apoptosis at 24 h to the initial concentration of active TGF-β1 in the cell culture or computer simulated model. The simulation output closely matches the experimental outputs.

The cellular scale ABM is calibrated to reflect *in vitro* experimental data generated by our group (Figures 4, 5). Fibroblast differentiation is calibrated to fit *in vitro* studies of fibroblast differentiation described above. We assume that the probability that a fibroblast will differentiate in a given simulation time step is linearly related to the amount of αSMA synthesized by the fibroblast:

(1) $$\begin{array}{cc} \begin{array}{l} Probability\;of\;differentiation=\\ \;slope\;*\;\frac{amount\;of\;\alpha SMA\;synthesized\;by\;the\;cell}{maximum\;amount\;of\;\alpha SMA\;a\;cell\;can\;synthesize} \end{array} & \left(1\right) \end{array}$$

where the slope determines the sensitivity of the system to αSMA. This relationship allows us to calibrate the slope and max αSMA so that the system reasonably fits experimental data of fibroblast differentiation (Figure 4). We also assume that the probability that an epithelial cell will apoptose in a given time step is proportional to the amount of TGF-β1 it has bound (Figure 5), and that this relationship is captured by a saturation curve:

(2) $$\begin{array}{cc} \begin{array}{l} Probability\;of\;apoptosis=\\ \;m\;*\;\frac{\begin{array}{l} amount\;of\;TGF\text{-}\beta \text{1}\\ \;bound\;by\;the\;cell \end{array}}{\begin{array}{l} \;amount\;of\;TGF\text{-}\beta 1\;bound\;by\;the\;cell\\ \;+\left(k\text{ * }amount\;PGE_{2}\;bound\;by\;the\;cell\right)+C \end{array}} \end{array} & \;(2) \end{array}$$

where *m* determines the sensitivity of the system to TGF-β1, *k* determines the sensitivity of the system to inhibition by PGE_2_, and *C* is a non-zero constant. We calibrate *m, k*, and *C* so that the system reasonably fits experimental data.

Initial conditions for the cellular scale virtual co-culture ABM are chosen to replicate experimental conditions. Five thousand epithelial cells and 500 fibroblasts are seeded on the surface of the model plate at time 0, creating a simulation environment that has ~84% cellular confluence. One thousand five hundred simulations (virtual experiments) were performed varying all parameter values by ~2 orders of magnitude except for the initial conditions and diffusivities of TGF-β1 and PGE_2_.

### Uncertainty and sensitivity analysis

We use uncertainty analysis to quantify how variation in parameter values leads to variation in model output (Marino et al., 2008). These variations can occur at the molecular and cellular scales in the model and can influence outputs spanning these biological scales. When parameters at one scale influence outcomes at that scale they are considered to have an intra-model influence. Intra-model influences can occur within either the molecular or cellular scale models. When mechanisms at one scale effect outcomes at the other scale these parameters are said to have inter-model influence. Uncertainty analysis allows us to observe model outcomes based on a wide value range for each parameter value. We vary numerous parameters in the model over a wide range (two orders of magnitude) and compare how these variations affect model outputs. Sensitivity analyses enable us to identify which model parameters have a significant influence and the extent of that influence on a given model output (Marino et al., 2008; Warsinske et al., 2015). Partial rank correlation coefficients (PRCCs) are used to determine the sensitivity of an output to a given parameter. PRCC values describe the correlation between the parameter value and the output in a range from −1 to +1. PRCC values are differentiated using a student *t*-test of significance. In this work we use the LHS algorithm to generate 500 unique parameter sets, and run each set in triplicate (Marino et al., 2008). PRCC values are considered significant and with a *p* < 0.01. We evaluate our model simulations (defined below) at day 7 because this time-point reflects the full range of possible model outcomes.

### Multi-scale model simulation

Our hybrid multi-scale model links both the molecular and cellular scale models, described previously. The combined model, resulting from the linking of the two models (see *Linking molecular and cellular scale models* above) allows us to simulate biological events with molecular and cellular scale details over time. Simulations are defined as an iteration of the multi-scale model that is run on the computer over a defined period of time. Simulations include a set of initial conditions, model rules, equations, and parameter values (see Supplementary Material for complete description of parameters and rules).

Model simulations can be evaluated by comparing individual outputs or the combined overall outcome of the simulation. Initial conditions include the number of each cell type, the concentrations of TGF-β1 and PGE_2_ in the environment, and the number of TGF-β1 receptors present on each fibroblast at time 0. In our model we track number of epithelial cells, fibroblasts, and myofibroblasts over time and position in the environment. Model outputs consist of not only these numbers in space and time but also concentrations of PGE_2_ and TGF-β1. A model outcome differs from a model output in that it encompasses multiple outputs and evaluates them over the entire timespan of the simulation. An example of a model outcome would be “*Rapid epithelial cell death with fibroblast proliferation and differentiation”* where outputs for all cell types are considered.

## Results

### TGF-β1 and PGE_2_ modulate fibroblast proliferation *in vitro* in a dose dependent manner

Previous work has demonstrated the roles of TGF-β1 and PGE_2_ in fibroblast to myofibroblast differentiation (Kolodsick et al., 2003; Thannickal et al., 2003; Epa et al., 2015; Warsinske et al., 2015). We sought to determine the capacity for TGF-β1 and PGE_2_ to influence IMR-90 fibroblast proliferation *in vitro*. We first compared levels of radioactive thymidine incorporation after 48 h in IMR-90 cultures with complete media (CM) or CM and the addition of 0.1–4.0 ng/ml acid activated TGF-β1 (Figure 3A). Data show no increase in proliferation and no decrease in proliferation in fibroblasts treated with 0.1, 0.5, or 1.0 ng of acid activated TGF-β1. A significant decrease in proliferation in 2.0 or 4.0 ng of TGF-β1 indicates decreased proliferation under these conditions (Figure 3A). We next compared levels of radioactive thymidine incorporation after 48 h of culture in CM alone, or CM and 0.1–100 nM of exogenous PGE_2_ (Figure 3B). Data show increased proliferation of fibroblasts in 0.1 nM PGE_2_. Fibroblasts cultured in CM with 1.0 nM PGE_2_ showed no change in proliferation. Data also show that 10 or 100 nM concentrations of PGE_2_ induced a significant decrease in proliferation (Figure 3B).

### TGF-β1 mediates fibroblast differentiation and epithelial cell death *in vitro* in a dose dependent manner that is captured by the multi-scale model

TGF-β1 has been demonstrated to induce fibroblast differentiation (Desmouliere et al., 1993; Kolodsick et al., 2003; Thannickal et al., 2003; Thomas et al., 2007; Epa et al., 2015). αSMA expression is a marker of fibroblast differentiation (Hinz et al., 2007). We sought to determine the ability of TGF-β1 to induce fibroblast differentiation by determining how an initial dose of TGF-β1 affects the proportion of differentiated fibroblasts at 24 h. We cultured fibroblasts in serum free media (SFM) alone, or SFM plus initial concentration of 0.1, 0.5, 1.0, 2.0, and 4.0 ng/ml exogenous acid-activated TGF-β1 for 24 h and counted the number of αSMA positive cells compared to the total number of cells present in high powered microscope fields (Figure 4). Data show a positive dose response between the concentration of initial active TGF-β1 and the proportion of activated fibroblasts at 24 h.

To capture this fibroblast dynamic in our computational model, we used Equation (1) to express the relationship between αSMA synthesis and fibroblast differentiation. We performed 1500 simulations each with 5000 fibroblasts and an initial concentration of 0.0–5.0 ng/ml exogenous acid-activated TGF-β1. After a simulated time of 24 h, we calculated the percent of αSMA positive cells in the total cell population (Figure 4). Our model captures the biology of fibroblast differentiation by recapitulating the dose response observed in the experimental dataset.

We sought to identify the correlation between TGF-β1 and epithelial cell death by determining how an initial dose of TGF-β1 affects the percentage of caspase positive epithelial cells at 24 h. TGF-β1 has been demonstrated to induce epithelial cell apoptosis (Bohm et al., 2014; Crosas-Molist and Fabregat, 2015) and caspase activation is a marker of apoptosis (Budihardjo et al., 1999). We cultured type II alveolar epithelial cells in SFM or SFM plus an initial concentration of 0.5, 1.0, 2.0, and 4.0 ng/ml exogenous acid-activated TGF-β1. At 24 h, we calculated the percentage of caspase positive cells in high-powered microscope fields (Figure 5). Data show a positive dose response between the concentration of initial active TGF-β1 and the proportion of apoptotic epithelial cells at 24 h.

To capture epithelial cell dynamics in our computational model, we used Equation (2) to express the relationship between TGF-β1 and epithelial cell apoptosis. We performed 1500 simulations each with 5000 epithelial cells and an initial concentration of 0.0–4.0 ng/ml exogenous acid-activated TGF-β1. After a simulated time of 24 h, we calculated the percent of the total population of dead epithelial cells. Our model captures the biology of epithelial cell apoptosis by recapitulating the dose response observed in the experimental dataset (Figure 5).

### Multi-scale model captures a wide range of possible fibroblast, myofibroblast, and epithelial cell outcomes in a virtual co-culture environment

**Figure 6:**
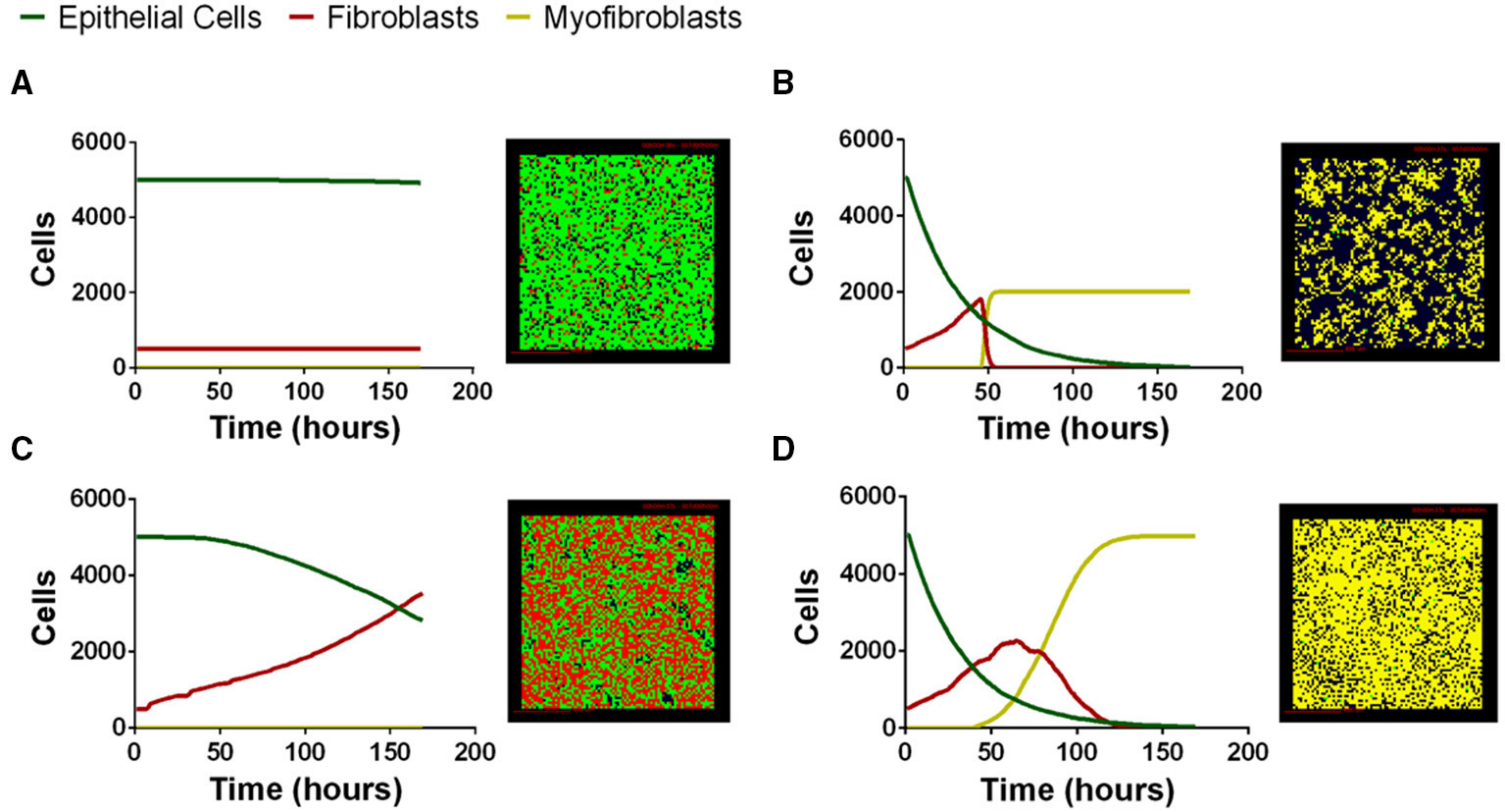
**Four distinct classes of multi-scale model simulation outcomes varying parameter values**. **(A)** *Healthy tissue outcome.* Complete epithelial cell survival can be achieved by baseline parameter values in the model. **(B)** *Rapid epithelial cell death with fibroblast proliferation and differentiation*. Parameter combinations leading to rapid epithelial cell death and early fibroblast differentiation include high TGF-β1 synthesis ($k_{syn}^{{TGF\beta 1}_{lat}}\;=$ 1.07 × 10^−16^). **(C)** *Gradual epithelial cell death with fibroblast proliferation*. Parameter combinations leading to gradual epithelial cell death include low PGE_2_ synthesis (*V*_*ePGE*2_ = 3.16 × 10^−27^) and low TGF-β1 proliferation threshold (*min*_*prolifTGFβ*1_ = 1.15 × 10^−25^). **(D)** *Excessive fibroblast proliferation and differentiation*. Parameter combinations leading to excessive fibroblast proliferation include high TGF-β1 synthesis ($k_{syn}^{{TGF\beta 1}_{lat}}\;=$ 9.86 × 10^−19^), high myofibroblast TGF-β1 binding (*k*_*onM*_ = 0.001, and decreased PGE_2_ synthesis *V*_*ePGE*2_ = 1.69 × 10^−29^). For complete parameter ranges please see Supplementary Material Tables S1–S4. For full length time-lapse simulations please see http://malthus.micro.med.umich.edu/lab/movies/Co-culture/.

We next tested whether our computational model can capture the range of possible biological co-culture outcomes observed experimentally. We explored outcomes for a range of physiologically plausible parameter values derived from literature, our work, or estimated using uncertainty analysis (Tables S1–S4); varying parameter values allows us to manipulate the relative importance of particular mechanisms in the model. Multi-scale model outcomes fell primarily into four categories that we classify as: *healthy tissue outcome, rapid epithelial cell death with fibroblast proliferation and differentiation, gradual epithelial cell death with fibroblast proliferation*, and *early epithelial cell death with excessive fibroblast proliferation and differentiation*. Representative simulations at the end of 7 days illustrate model outcomes (Figure 5). We have selected four outcomes that are representative of common trends identified in our LHS. Outcomes falling into each category share some parameter trends. These trends are described in the figure legend with specific values given for the simulation shown (see Supplementary Material Tables S1–S4 for full parameter ranges). Our first outcome category, a healthy tissue outcome, shows that under idealized conditions epithelial cells survive and fibroblasts remain quiescent (Figure 6A). By the end of 7 days fibroblasts had not proliferated and the epithelial cells survived. Our second outcome category, rapid epithelial cell death with fibroblast proliferation and differentiation, shows that under some conditions epithelial cells undergo rapid apoptosis (Figure 6B); ~50% of the epithelial cells died in the first 24 h. Fibroblast proliferation occurs during the first 24 h. Fibroblast to myofibroblast differentiation begins very early in the simulation. By 24 h all fibroblasts have differentiated restricting proliferation and resulting in fewer cells overall. In the third outcome category, gradual epithelial cell death with fibroblast proliferation, fibroblasts undergo a large amount of proliferation more than doubling in the first 48 h of the simulation (Figure 6C). In this simulation epithelial cells experience gradual cell death, and by 168 h nearly half of the epithelial cells have died. Our fourth outcome category, excessive fibroblast proliferation, and differentiation, shows nearly complete epithelial cell death by 168 h (Figure 6D). In this simulation fibroblasts proliferate rapidly doubling by 48 h. At this time some fibroblasts begin to undergo differentiation while others continue to proliferate. After 168 h all of the fibroblasts have differentiated so that the entire plate is covered with myofibroblasts (Figure 6D).

### Analysis of the multi-scale model reveals key mechanisms driving fibroblast proliferation, differentiation, and epithelial cell survival in a co-culture environment

A wide range of possible multi-scale model simulation outcomes for fibroblast proliferation, differentiation, and epithelial cell survival can be achieved by manipulating mechanisms of fibroblast and epithelial cell co-regulation (Figure 6). To determine which mechanisms and to what extent they are responsible for driving these different outcomes, we performed sensitivity analysis on the outputs from 1500 simulations of our multi-scale model at day 7 as described in Methods. The analysis predicts mechanisms dictating fibroblast proliferation without differentiation (determined by fibroblast number) (Table 1A). Of the mechanisms found to drive fibroblast proliferation, PGE_2_ synthesis had the strongest influence (*p* < 0.01). The analysis predicts which mechanisms dictate myofibroblast differentiation (determined by number of myofibroblasts) (Table 1B). Of these mechanisms, TGF-β1 synthesis and PGE_2_ inhibition of TGF-β1-induced differentiation had the strongest effect on myofibroblast number (*p* < 0.01). PRCC analysis predicts that the strongest mechanisms driving epithelial cell survival (determined by epithelial cell number) (Table 1C) are predominantly different from mechanisms driving fibroblast proliferation (Table 1A) and differentiation (Table 1B). Of the mechanisms found to drive epithelial cell survival; TGF-β1 synthesis and TGF-β1 activation had the strongest effect on epithelial cell number (*p* < 0.01). These mechanisms may be strong candidates for therapeutic intervention.

**Table 1:** **Primary mechanisms driving fibroblast, myofibroblast, and epithelial cell numbers**.

**(A) Significant PRCC values for fibroblast number at day 7 (*****p*** < 0.01**)**					
TGF-β1 synthesis	PGE2 binding rate	Fibroblast sensitivity to PGE2	Fibroblast insensitivity to TGF-β1	TGF-β1 proliferation threshold	PGE2 proliferation maximum
−0.11	−0.17	−0.12	0.12	−0.11	0.14
Probability of fibroblast movement	αSMA differentiation maximum	TGF-β1 to PGE2 differentiation threshold	PGE2 synthesis	Epithelial cell sensitivity to PGE2	
−0.08	0.07	0.19	−0.40	0.07	
**(B) Significant PRCC values for myofibroblast number at day 7 (*p* < 0.01)**					
TGF-β1 activation	TGF-β1 synthesis	Fibroblast insensitivity to TGF-β1	PGE2 inhibition of TGF-β1 induced differentiation	PGE2 synthesis	Epithelial cell TGF-β1 binding rate
0.10	0.54	−0.13	−0.26	−0.14	−0.09
**(C) Significant PRCC values for epithelial cell number at day 7 (*p* < 0.01)**					
TGF-β1 receptor dissociation	TGF-β1 synthesis	TGF-β1 activation	PGE2 binding rate	Fibroblast insensitivity to TGF-β1	αSMA differentiation maximum
−0.10	−0.79	−0.47	0.11	−0.14	−0.09

### Multi-target intervention strategies promote healthy tissue repair better than single target strategies

From our sensitivity analysis, we established a list of the primary mechanisms driving fibroblast, myofibroblast, and epithelial cell outcomes (Table 1). Some of these mechanisms reiterate previous understandings about fibrosis, and others provide new insight. Current therapies for pulmonary fibrosis target TGF-β1 signaling in order to prevent fibroblast dysregulation (King et al., 2014; Xaubet et al., 2014; Myllarniemi and Kaarteenaho, 2015). Our analysis identifies that inhibiting TGF-β1 receptor/ligand complex internalization, the first step in TGF-β1 signaling, results in an overall decrease in myofibroblast number (Table 1B). *In vitro* studies show that high levels of PGE_2_ inhibit fibroblast proliferation (Figure 3). Our sensitivity analysis further emphasizes this relationship by highlighting the strong negative correlation between fibroblast number and epithelial cell synthesis of PGE_2_ (Table 1A).

**Figure 7:**
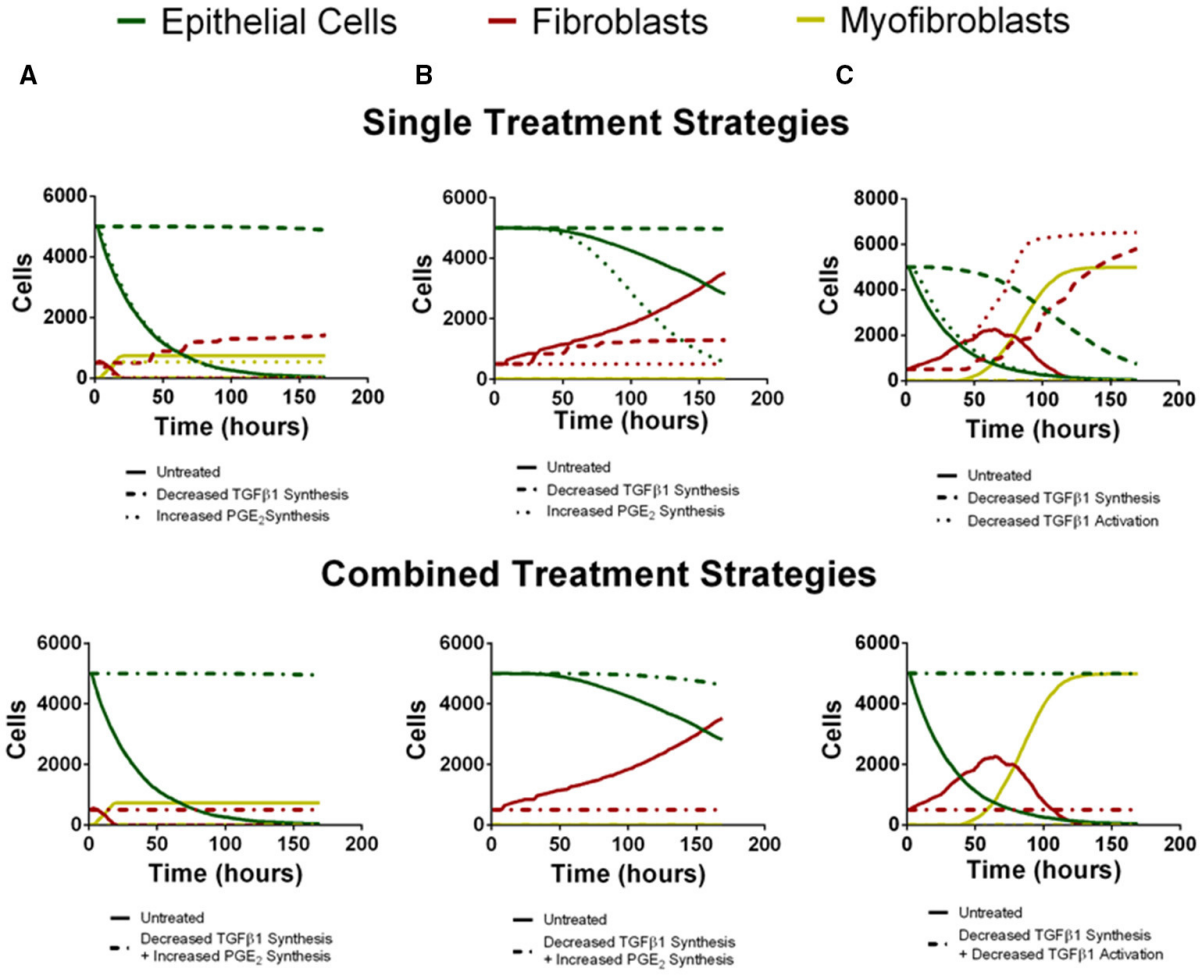
**Virtual individual and combined treatment outcomes for three case studies (compare to B,C,D from Figure 6)**. Green line represents epithelial cells, red line represents fibroblasts and yellow line represents myofibroblasts. **(A)** *Rapid epithelial cell death and recovery*. The top panel depicts untreated simulation (solid line) decreased TGF-β1 synthesis (dashed line) or increased PGE_2_ synthesis (dotted line) in isolation. Decreased TGF-β1 synthesis preserves epithelial cell number but does not inhibit fibroblast proliferation. Increased PGE_2_ synthesis inhibits excessive fibroblast proliferation but does not rescue epithelial cell number. The bottom panel depicts untreated simulation (solid line) and decreased TGF-β1 synthesis in combination with increased PGE_2_ synthesis (dot-dashed line). Combined treatment restricts fibroblast proliferation and preserves epithelial cell survival. **(B)** *Gradual Epithelial cell death and recovery*. The top panel depicts untreated simulation (solid line) decreased TGF-β1 synthesis (dashed line) or increased PGE_2_ synthesis (dotted line) in isolation. Decreased TGF-β1 synthesis preserves epithelial cell number but does not inhibit fibroblast proliferation. Increased PGE_2_ synthesis inhibits excessive fibroblast proliferation but results in more rapid decline in epithelial cell number. The bottom panel depicts untreated simulation (solid line) and decreased TGF-β1 synthesis in combination with increased PGE_2_ synthesis (dot-dashed line). Combined treatment restricts fibroblast proliferation and preserves epithelial cell survival. **(C)** *Excessive fibroblast proliferation and recovery*. The top panel depicts untreated simulation (solid line) decreased TGF-β1 synthesis (dashed line) or decreased TGF-β1 activation (dotted line) in isolation. Decreased TGF-β1 synthesis increases but does not preserve epithelial cell number and does not inhibit fibroblast proliferation. Decreased TGF–β1 activation does not inhibit excessive fibroblast proliferation or rescue epithelial cell number The bottom panel depicts untreated simulation (solid line) and decreased TGF-β1 synthesis in combination with or decreased TGF-β1 activation (dot-dashed line). Combined treatment restricts fibroblast proliferation and preserves epithelial cell survival.

To predict the success of potential therapeutic strategies, we simulated therapeutic interventions by either promoting or inhibiting the mechanisms identified by our sensitivity analysis as the most significant for driving fibroblast proliferation, fibroblast differentiation, and epithelial cell survival. We simulated these therapeutic strategies in the three outcome categories identified as rapid epithelial cell death with fibroblast differentiation, gradual epithelial cell death with fibroblast proliferation and differentiation, and excessive fibroblast proliferation and differentiation (Figure 7). We first tested single intervention strategies intended to either inhibit fibroblast proliferation or differentiation, or to promote epithelial cell survival for each outcome category. We then took a two-hit approach and combined treatment strategies that inhibit fibroblast proliferation or differentiation together with strategies that promote epithelial cell survival. Thus, for each case we tested two interventions independently (Figure 7 top) and then in combination (Figure 7 bottom).

For the outcome category rapid epithelial cell death with fibroblast differentiation (Figure 7A), we virtually reduced fibroblast synthesis of TGF-β1 or we increased epithelial cell synthesis of PGE_2_. Decreasing TGF-β1 synthesis alone inhibits fibroblast to myofibroblast differentiation and rescues the epithelial cells at 168 h but due to the presence of low levels of TGF-β1 fibroblast proliferation is not inhibited. Increased synthesis of PGE_2_ inhibited fibroblast proliferation but did not decrease the rate of epithelial cell death (Figure 7A top). Simultaneously decreasing TGF-β1 synthesis together and increasing PGE_2_ synthesis inhibited fibroblast to myofibroblast differentiation and also restored epithelial cell survival by day 7 (Figure 7A bottom).

For the outcome category, gradual epithelial cell death with fibroblast proliferation (Figure 7B), we virtually reduced fibroblast synthesis of TGF-β1 or we increased the rate of PGE_2_ synthesis by epithelial cells. Reducing TGF-β1 synthesis alone rescues the epithelial cells at 168 h but it does not inhibit fibroblast proliferation. Increasing PGE_2_ synthesis inhibited fibroblast to myofibroblast differentiation and fibroblast proliferation. Increased PGE_2_ synthesis improved epithelial cell survival by day 7 but not to the same level as decreasing TGF-β1 synthesis (Figure 7B top). Combining these effects both inhibited fibroblast proliferation and differentiation while fully restoring epithelial cell survival by day 7 (Figure 7B bottom).

Finally, for the outcome category, excessive fibroblast proliferation, and differentiation, we virtually reduced TGF-β1 synthesis or reduced TGF-β1 activation. Reducing TGF-β1 synthesis alone inhibited fibroblast to myofibroblast differentiation and made a dramatic improvement in epithelial cell survival at day 7, but did not inhibit fibroblast proliferation. Decreasing TGF-β1 activation had almost no effect on epithelial cell survival, strongly inhibited fibroblast differentiation, but showed no ability to inhibit fibroblast proliferation (Figure 7C top). However, combining these effects restored epithelial cell survival by day 7 while inhibiting fibroblast proliferation and differentiation (Figure 7C bottom). *A conclusion from the model is that different mechanisms must be targeted simultaneously to affect outcomes of epithelial cell survival, fibroblast proliferation, and myofibroblast differentiation.*

## Discussion

Pulmonary fibrosis results from dysregulation of the wound healing process in the lungs. Epithelial cells, fibroblasts, and myofibroblasts each play key roles in tissue regeneration after injury. The actions of these different cell types are regulated by pro- and anti-fibrotic cytokines such as TGF-β1 and PGE_2_ respectively. During fibrosis, excessive fibroblast proliferation/accumulation and differentiation into myofibroblasts paired with epithelial cell death results in thick, stiff, and scarred lung tissue that is not suitable for breathing and gas exchange. Our analysis allowed us to identify key mechanisms driving fibroblast/myofibroblast dysregulation and epithelial cell death. With this analysis we can predict potential therapeutic targets and strategies for the treatment of pulmonary fibrosis.

The use of *in silico* methods in tandem with experimental approaches allows us to identify relationships between specific mechanisms contributing to fibroblast and epithelial cell co-regulation and their outcomes such as epithelial cell survival and fibroblast proliferation and differentiation during fibrosis. Our multi-scale, hybrid ABM is a tool that simulates a computation platform similar to an *in vitro* co-culture system. To ensure that our model accurately reflects biology we used data from the literature or generated it herein to calibrate it. We take a reductionist approach to understanding fibroblast differentiation and epithelial cell regulation and do not capture the full complexity of the lung environment. Nevertheless, like an *in vitro* culture system, our model allows us to test specific mechanisms involved in fibroblast/epithelial cell co-regulation. With this unique tool we can evaluate all parts of the co-regulatory system simultaneously at multiple biological scales over time. Within our multi-scale model simulations we can vary rates and magnitudes of cellular and chemical interactions across a wide range, simulating the effects of thousands of theoretical intervention strategies. The information produced by these simulations includes cell number, local and total chemical concentrations, numbers of proliferation or apoptosis events within a given time frame, and many other details about the simulated cells and environment. Analysis of these simulated outputs allows us to determine which potential interventions or combinations of interventions promote epithelial cell survival and inhibit dysregulation of fibroblasts and myofibroblasts.

Our simulations yield four major categories of outcomes characterized by the number of cells left in each cell class after 7 days. We describe these common cases as healthy tissue outcome, rapid epithelial cell death with fibroblast proliferation and differentiation, gradual epithelial cell death with fibroblast proliferation, and excessive fibroblast proliferation and differentiation. It should be clear that there is a wide range of outcomes in between these four classes, as cell behavior in our model and in reality is not discrete but a continuous range of possibilities. Our results hold nonetheless. Through uncertainty and sensitivity analysis, we identify mechanisms driving fibroblast, myofibroblast, and epithelial cell outcomes defined by cell numbers at 7 days. Although several mechanisms have a significant impact on fibroblast cell number, the strongest regulator is PGE_2_ synthesis by epithelial cells. For myofibroblasts, TGF-β1 synthesis and the ratio of TGF-β1 to PGE_2_ permissive for differentiation are the key mechanisms driving cell number. Epithelial cell outcome is most dependent on TGF-β1 synthesis and TGF-β1 activation. It is important to note that fibroblast, myofibroblast and epithelial cell survival outcomes are largely affected by different mechanisms. This implies that treatment strategies intended to reduce or reverse tissue damage associated with fibrosis need to target multiple mechanisms specific to different cell types.

Further exploration of this “two-hit” therapeutic approach emphasizes the efficacy of combinatorial treatment strategies over single target strategies. We performed rescue experiments on the three previously identified poor model outcome categories. We reduced the synthesis of TGF-β1, increased the synthesis of PGE_2_, and or decreased TGF-β1 activation in these cases and found that in each case, a two-hit treatment strategy was more effective than a single target approach. In most cases, one intervention targeted epithelial cells and the other targeted fibroblasts and/or myofibroblasts. In some cases there were overlapping effects on multiple cell types, but in all cases a minimum of two strategies were required for a complete rescue effect.

As previously described, there are only two therapeutics available for the treatment of pulmonary fibrosis in the United States; Nintedanib and Pirfenidone. Independently these drugs each target one aspect of fibrotic dysregulation. Combined they could potentially have a synergistic effect. While it is interesting to speculate on the biologic usefulness of this approach, currently the costs associated specifically with Pirfenidone and Nintedanib would be prohibitive for most patients. However, our model predicts that therapeutic strategies addressing multiple aspects of fibrotic disease are essential for the effective treatment of pulmonary fibrosis, and previous analyses have suggested that single therapeutic strategies are insufficient (Kendall and Feghali-Bostwick, 2014). We must identify combined therapeutics strategies that are not cost prohibitive to improve the prognosis of pulmonary fibrosis patients.

Other existing therapeutic strategies that could be considered for a more cost effective combined treatments include, but are not limited to, TGF-β1 receptor fusion proteins (Muraoka et al., 2002), recombinant human IL-1α and/or TNFα, arachidonic acid (Chen et al., 1995), and IL-13 receptor inhibitors (Hashimoto et al., 2001; Kraft et al., 2001; Saito et al., 2003) (Table 2). When considering inhibition of TGF-β1 signaling as a therapeutic, one must be cautious of the fact that TGF-β1 is a potent regulator of autoimmunity (Letterio and Roberts, 1998) and global inhibition of TGF-β1 receptor signaling could result in the development of profound immune activation and systemic autoimmunity. Thus, localized delivery to the lung and perhaps more targeted approaches to block downstream mediators may show better efficacy overall (Cilfone et al., 2015c).

**Table 2:** **Potential therapeutics for key mechanisms of fibrosis**.

**Mechanism**	**Influence on cell number**	**Therapeutic**	**References**	**Influence on mechanism**
**FIBROBLASTS**				
TGF-β1 synthesis	↑	Pirfenidone	Dosanjh et al., 2002	↓
PGE2 synthesis	↓	IL-1α, TNFα, AA, IL-13 receptor antagonist	Chen et al., 1995; Hashimoto et al., 2001; Kraft et al., 2001; Saito et al., 2003	↑
PGE2 binding	↓	CP-533536	Li et al., 2003	↑
PGE2 sensitivity	↓	Forskolin		↑
Proliferation	↑	Nintedanib, IL-13 receptor antagonist (QAX 576)	Hashimoto et al., 2001; Kraft et al., 2001; Saito et al., 2003; Loomis-King et al., 2013; Myllarniemi and Kaarteenaho, 2015	↓
**EPITHELIAL CELLS**				
TGF-β1 synthesis	↓	Pirfenidone	Dosanjh et al., 2002	↓
TGF-β1 activation	↓	anti-αVβ6	Puthawala et al., 2008	↓
TGF-β1 sensitivity	↓	TGF-β1 receptor fusion, TGF-β1 receptor antagonist (A8301)	Muraoka et al., 2002; Prasanphanich et al., 2014; Overgaard et al., 2015	↓

↑ Indicates promotes cell number or mechanism, ↓ Indicates restricts cell number or mechanism.

Recombinant human IL-1α and recombinant human TNFα have been shown to stimulate PGE_2_ synthesis by endometrial cells via the up regulation of the rate-limiting cyclooxygenase-2 (COX-2) enzyme important for conversion of arachindonic acid to PGH_2_ (Chen et al., 1995) and ultimately PGE_2_. By mimicking PGE_2_ binding, a mechanism highlighted by our sensitivity analysis, cAMP analogs could limit fibroblast activation, and also promote epithelial cell survival. One of the challenges with PGE_2_-based therapeutics are that lipid mediators have short half-lives *in vivo*, and systemic delivery of PGE_2_ could result in hemodynamic complications due to signaling via other EP receptors in multiple cell types (Audoly et al., 1999; Sugimoto and Narumiya, 2007).

IL-13 has been show to promote fibroblast proliferation and increase αSMA synthesis, ultimately leading to differentiation (Hashimoto et al., 2001; Kraft et al., 2001; Saito et al., 2003). Reduction of IL-13 signaling in fibroblasts through receptor inhibitors such as QAX 576 (Novartis) (Loomis-King et al., 2013) could reduce fibroblast cell number, differentiation, and tissue remodeling functions. It has also been hypothesized that IL-13 may reduce PGE_2_ production (Saito et al., 2003). Thus, inhibition of IL-13 receptor signaling may have a dual effect of decreasing fibroblast proliferation and increasing PGE_2_ synthesis.

In simulated combinations targeting fibroblasts and promoting epithelial cells, these therapeutics and others have the potential to provide synergistic improvements in outcomes over individual treatment strategies (Table 2). Fibroblasts, myofibroblasts, and epithelial cells have distinct regulatory mechanisms during wound healing. The dysregulation of any or all of these processes requires a multifaceted approach for the full restoration of tissue integrity. Addressing epithelial cell survival in conjunction with solving fibroblast dysregulation using either new or already available therapeutics is the necessary next step to providing therapy for pulmonary fibrosis.

## Author contributions

The authors listed made a major contribution to the conception or design of the work (HW, JL, BM, DK), data collection (HW, AW, BM), data analysis and interpretation (HW, BM, DK), drafting of the manuscript (HW, DK), critical revision of the manuscript (HW, KK, JL, BM, DK), and final approval of the version to be published (HW, AW, KK, JL, BM, DK).

### Conflict of interest statement

The authors declare that the research was conducted in the absence of any commercial or financial relationships that could be construed as a potential conflict of interest.
