# Supplementary material for: Computational Modeling Predicts Simultaneous Targeting of Fibroblasts and Epithelial Cells Is Necessary for Treatment of Pulmonary Fibrosis
Source: Front Pharmacol. 2016 Jun 23;7:183. doi: 10.3389/fphar.2016.00183 (PMC4917547; doi:10.3389/fphar.2016.00183)
Supplement: Supplementary file 1 [file DataSheet1.DOCX]

***Supplimentary Material***

Computational modeling predicts simultaneous targeting of fibroblasts and epithelial cells is necessary for treatment of pulmonary fibrosis

Hayley C. Warsinske^1^, Amanda Wheaton^2^, Kevin Kim^2^, Jennifer J. Linderman^3^, Bethany B. Moore^1,2^, Denise E. Kirschner^1^*

1. Department of Microbiology and Immunology, University of Michigan Medical School, Ann Arbor, Michigan, United States of America
2. Department of Internal Medicine, University of Michigan Medical School, Ann Arbor, Michigan, United States of America
3. Department of Chemical Engineering, University of Michigan, Ann Arbor, Michigan, United States of America

Multi-Scale Model Rules

Here we describe the overall structure of our multi-scale hybrid agent-based model of a fibroblast/epithelial cell co-culture system. There are two key models that are linked to create the multi-scale model: *a cellular scale* model and a *molecular scale* model. Here we include a detailed description of the stochastic processes occurring in the cellular scale model that reads out at a tissue scale. The discrete molecular scale processes are described in full detail in a previous study ([1](#_ENREF_1)). A schematic capturing how these models were derived independently and are linked is included in the main text (Figure 2) along with a description of how the model linking is done (see Methods). All values and definitions of parameter that are used in the models are listed in the Supplementary Tables S1, S2, S3, S4.

**Overall Structure of the Cellular Scale ABM**

Agent based models are designed focusing on four components as follows: an environment (simulated tissue culture plate), agents (fibroblasts, myofibroblasts, and epithelial cells), rules that govern agent behavior, and a time-step that dictates how frequently events in the model are updated (Δt).

***Environment:*** The three-dimensional environment in this model represents one tenth of a well in a 96 well tissue culture plate. At the bottom of the model environment is a square surface measuring 1.78mm x 1.78mm. This surface represents the plastic at the bottom of a dish. The plate is divided into a 6561 compartment grid (81 compartments by 81 compartments). Each compartment is a 22µm x 22μm x 22μm cube. Cellular agents are adherent to the “plastic” and are therefore restricted to the grid compartments directly above the *in silico* plastic bottom surface in what will be defined as the cellular layer. Because cells are adhesion dependent, cells can only move in two dimensions within the cellular layer. We assume that each grid compartment can contain only one agent at a time based on their size. Their movement is guided by the ABM rules defined below. Above this layer is a three-dimensional layer that represents the volume of the plate (81 compartments by 81 compartments by 281 compartments). The full 3D model contains 282 layers each with 6561 compartments. All layers above the cellular layer represent *in silico* media in the well of a tissue culture plate.

Some mediators are restricted to the two-dimensional (2D) bottom surface layer while others can diffuse throughout the three dimensional compartments. Extra-cellular matrix (ECM) proteins are secreted directly only the surface of the virtual dish where they are cross-linked into a matrix. These proteins accumulate on this layer and do not diffuse. Latent TGF-β1 (as secreted by fibroblasts and myofibroblasts) is secreted into the extracellular matrix where it adheres until the protein is activated. Proteins that do not diffuse are stored as continuous values in the compartment where they are secreted. Activated TGF-β1 and PGE_2_ are soluble mediators which can diffuse in three dimensions throughout the entire volume of the model, including the cellular layer and all media layers above it. These mediators are also stored as continuous values that occupy space anywhere within the model.

We built an 81 x 81 x 282 3D grid. We assume there are no flux boundary conditions for all boundaries of the model grid for all agent movement and molecular diffusion steps. 5000 epithelial cells and 500 fibroblasts are randomly placed on the grid, each within its own compartment in the cellular layer. This is consistent with *in vitro* studies typically plating a 10:1 ratio of epithelial cell to fibroblasts ([2](#_ENREF_2)).

***Agents****:* There are two distinct types of cellular agents in the model, fibroblasts (representing pulmonary fibroblasts) and epithelial cells (representing alveolar epithelial cells). We assume that fibroblasts can differentiate into myofibroblasts, a process that is driven by αSMA synthesis (see Methods in main text). We also assume they do not de-differentiate back into a fibroblast, but rather remain a myofibroblast for the duration of a simulation. Once differentiated, we assume that myofibroblasts can no longer move or proliferate. Myofibroblasts are also susceptible to undergoing apoptosis in a probabilistic fashion.

***Cellular Scale ABM Rules***: ABM rules define cellular interactions as well as other processes in the model including cell movement, proliferation, death, and mediator secretion according to known biological behaviors in vitro. Additional rules in the model define molecule diffusion and degradation based on known rates identified in the literature (see Parameters below).

**1. Cell Movement*.*** We assume that fibroblasts are the only cellular agents with the ability to move. They can move in 8 possible directions within the cellular layer. Fibroblasts examine their Moore neighborhood (the nine grid compartments surrounding the cell including the compartment occupied by the cell) and determine whether there is an unoccupied compartment. If an unoccupied compartment is found, then the cell has a probability of moving into that compartment (Table S2). A fibroblast can move no more than once during a single model time step.

**2. Cell death, proliferation and differentiation*. A.*** *Cell death due to age.* Two agent types, myofibroblasts and epithelial cells, can die of old age. When a cell reaches its maximum allowable age there is a probability that the cell will die. Dead cells will be removed from the grid (Table S1, S3). ***B.*** *Epithelial cell apoptosis due to TGF-β1 concentration.* Epithelial cells are sensitive to high concentrations of TGF-β1. At birth each epithelial cell is assigned a maximum TGF-β1 threshold from a uniform distribution. This threshold captures the idea that that each epithelial cell has a slightly different sensitivity to TGF-β1 ([3](#_ENREF_3)). At each model time step, epithelial cells checks the cumulative amount of TGF-β1 that it has bound. If the cumulative bound TGF-β1 reaches or exceeds the cells maximum threshold, then the cell dies and is removed from the grid. ***C.*** *Fibroblast proliferation.* Four rules govern when a fibroblast can proliferate. (1) It must be 24 hours since the cell last divided ([4](#_ENREF_4)). (2) The cell must have bound enough TGF-β1 to meet the TGF-β1 proliferation threshold (Table S1). (3) The cell must not have bound more than the maximum amount of PGE_2_ permissive of proliferation (Table S1). (4) There must be an empty compartment in the fibroblast’s Moore neighborhood for a daughter cell to enter ([5](#_ENREF_5), [6](#_ENREF_6)). If these four conditions are met, then the fibroblast has a non-zero probability of proliferating (Table S1). ***D.*** *Fibroblast to myofibroblast differentiation.* We assume that fibroblast to myofibroblast differentiation is determined by the amount of αSMA synthesized by a fibroblast and is described in detail in the “Multi-scale Model Calibration” section of the main text.

**3. Mediator Secretion**. We assume that fibroblasts are able to secrete both TGF-β1 and PGE_2_ at rates listed in Table S1, respectively. We also assume that epithelial cells secrete only PGE_2_ at rates listed in Table S4. Finally, we assume that myofibroblasts secrete ECM proteins with rates listed in Table S3.

**4. Diffusion and degradation**. Continuous molecular diffusion is calculated in three dimensions using the Fast Fourier Transform method ([7](#_ENREF_7)). To integrate this numerical method into the framework of an ABM we use the methods described in detail by Cilfone et al. ([8](#_ENREF_8)). Active TGF-β1 and αSMA model have degradation rate constants (Table S1). Latent TGF-β1 is stabile throughout the duration of the simulations in this paper.

***Time step:*** Interactions between agents are updated for each ABM time step (Δt = 1 hour) and are described above. Molecular scale processes, specifically TGF-β1 receptor ligand signaling in fibroblasts and secretion of mediators by all agent types, generally occur more quickly than cellular scale processes. For example, fibroblast proliferation takes about 24 hours ([9](#_ENREF_9), [10](#_ENREF_10)) where TGF-β1 receptor synthesis is around 4 minutes ([11](#_ENREF_11)). These molecular processes are therefore updated on a more frequent molecular time step (Δt = 10 seconds), and thus each molecular process is updated 360 times per ABM time step. Diffusion of soluble molecules is updated at the diffusion time step (Δt = 60 seconds).

***Simulation***: Our combined model is implemented in C++ and runs on Linux/MacOS/Windows. Documentation and pseudo code are available in the online Supplement. A Runge-Kutta 4 method is used to solve the ODEs. Each time step of the ABM simulation is further divided into 60 pieces (step size of 60s) to reduce error. Fast Fourier Transform and forward Euler methods are used to solve the chemical diffusion equation ([8](#_ENREF_8)) The molecular sub-model takes less than 0.1 second of real time each iteration of the molecular dynamics model ([12](#_ENREF_12)). Each simulation describing 7 days of co-culture takes 40 to 50 minutes to run on a 300 core computer cluster.

Multi-Scale Model Parameters

**Table S1: Extracellular mediator Parameters and model simulation times**

| *Parameter* | *Definition* | *Value* | *Source* |
| --- | --- | --- | --- |
| ${\Delta t}_{ABM}$ | Time over which the ABM is updated | 1 h | N/A |
| ${\Delta t}_{diffusion}$ | Time over which diffusion is solved | 60 s | N/A |
| ${\Delta t}_{smoother}$ | Time over which diffusion smoother is solved | 0.1 s | N/A |
| $N_{smoother}$ | Number of times diffusion smoother is solver in one diffusion time step | 10 | N/A |
| ${\Delta t}_{molecular}$ | Time over which TGF-β1 receptor ligand molecular model is solved | 10 s | N/A |
| $D_{TGF\beta1}$ | Diffusivity of active TGF-β1 | 2.7x10^-7^ cm^2^/s | DMW |
| $D_{PGE2}$ | Diffusivity of PGE_2_ | 5.23x10^-6^ cm^2^/s | DMW |
| $\delta_{TGF\beta1}$ | Active TGF-β1 degradation rate constant | [0.001 , 0.1] pM/min | ([13](#_ENREF_13)) |
| $\delta_{PGE2}$ | PGE_2_ degradation rate constant | [0.001 , 0.1] pM/min | Est. |

N/A = not applicable DMW = derived from molecular weight Est. = estimated by uncertainty and sensitivity analysis

**Table S2: Fibroblast Parameters**

| *Parameter* | *Definition* | *Value* | *Source* |
| --- | --- | --- | --- |
| $t_{proliferation}$ | Minimum time between proliferation events | 24 h | DNS |
| ${max}_{prolifPGE2}$ | Maximum bound PGE_2_ permissive of proliferation | [5x10^-21^, 1x10^-9^] pM | Est., Fig 3B |
| ${min}_{prolifTGF\beta1}$ | Minimum bound TGF-β1 necessary for proliferation | [1x10^-16^, 1x10^-14^] pM | Fig. S |
| $m_{differentiation}$ | Slope of the linear regression dictating fibroblast differentiation | 0.5 | Est. |
| $P_{move}$ | Probability of fibroblast movement | [1x10^-2^, 1] | N/A |

DNS = data not shown N/A = not applicable Est. = estimated by uncertainty and sensitivity analysis

**Table S3: Myofibroblast Parameters**

| *Parameter* | *Definition* | *Value* | *Source* |
| --- | --- | --- | --- |
| $V_{ECM}$ | Rate of ECM synthesis | [1, 100] units | Est. |
| ${min}_{age}$ | Minimum lifespan of myofibroblasts | 1440 h | ([14](#_ENREF_14)) |
| $P_{death}$ | Probability of death | [0, 1] | N/A |
| ${max}_{ECM}$ | Maximum amount of ECM in a single compartment | 100 units | N/A |

Est. = estimated by uncertainty and sensitivity analysis N/A = not applicable

**Table S4: Epithelial Cell Parameters**

| *Parameter* | *Definition* | *Value* | *Source* |
| --- | --- | --- | --- |
| $V_{ePGE2}$ | Rate of PGE2 synthesis by epithelial cells | [1x10^-20^, 1x10^-18^] pM/s |  |
| $k_{onE}$ | Rate of TGF-β1 binding by epithelial cells | [0.0009, 0.09] pM/s | Est. |
| $k$ | Magnitude of PGE2 protection from TGF-β1 induced apoptosis | [0.0001, 100/0] | Est. |
| $C$ | Non-zero constant | 7x10^-14^ | Est. |

Est. = estimated by uncertainty and sensitivity analysis

**Table S5A: Significant PRCC Values for Cell Number at Day 1 (p<0.01)**

|  | *TGF-β1 Activation* | *TGF-β1 Receptor Internalization Rate* | *TGF-β1 Receptor Recycling Rate* | *TGF-β1 Synthesis* | *TGF-β1 Receptor Degradation Rate* | *Latent TGF-β1 Degradation Rate* |
| --- | --- | --- | --- | --- | --- | --- |
| Fibroblast |  |  |  | 0.18 |  |  |
| Myofibroblast | 0.10 |  |  | 0.44 | -0.09 |  |
| Epithelial Cell | -0.65 | -0.10 | -0.08 | -0.87 |  | -0.07 |
|  | *PGE2 Binding Rate* | *Fibroblast Sensitivity to PGE2* | *Fibroblast Insensitivity to TGF-β1* | *TGF-β1 Proliferation Threshold* | *PGE2 Proliferation Maximum* | *αSMA Differentiation Maximum* |
| Fibroblast | -0.18 | 0.09 | 0.12 | -0.12 | 0.17 | 0.10 |
| Myofibroblast |  |  | -0.11 |  | -0.13 |  |
| Epithelial Cell | 0.11 |  | -0.15 |  | -0.07 |  |
|  | *TGF-β1 to PGE2 Differentiation Threshold* | *PGE2 Synthesis* | *Epithelial Cell TGF-β1 Binding Rate* |  |  |  |
| Fibroblast | 0.18 | -0.45 | -0.07 |  |  |  |
| Myofibroblast | -0.23 | -0.09 |  |  |  |  |
| Epithelial Cell |  |  |  |  |  |  |

**Table S5B: Significant PRCC Values for Cell Number at Day 2 (p<0.01)**

|  | *TGF-β1 Activation* | *Active TGF-β1 Degradation Rate* | *TGF-β1 Receptor Dissociation* | *TGF-β1 Receptor Internalization Rate* | *TGF-β1 Receptor Recycling Rate* | *TGF-β1 Synthesis* |
| --- | --- | --- | --- | --- | --- | --- |
| Fibroblast |  |  |  |  |  |  |
| Myofibroblast |  | 0.07 | 0.08 |  |  | 0.09 |
| Epithelial Cell | -0.07 |  | -0.08 | -0.07 | -0.09 |  |
|  | *TGF-β1 Receptor Degradation Rate* | *Latent TGF-β1 Degradation Rate* | *PGE2 Binding Rate* | *Fibroblast Sensitivity to PGE2* | *Fibroblast Insensitivity to TGF-β1* | *TGF-β1 Proliferation Threshold* |
| Fibroblast | 0.10 |  |  | -0.16 | 0.09 | 0.11 |
| Myofibroblast | 0.48 | -0.086 |  |  |  | -0.12 |
| Epithelial Cell | -0.86 |  | -0.60 | 0.10 |  | -0.15 |
|  | *PGE2 Proliferation Maximum* | *TGF-β1 to PGE2 Differentiation Threshold* | *αSMA Differentiation Maximum* | *TGF-β1 to PGE2 Differentiation Threshold* | *PGE2 Inhibition of TGF-β1 Induced Differentiation* | *Myofibroblast TGF-β1 Binding Rate* |
| Fibroblast | -0.11 | 0.15 |  | 0.09 | 0.17 | 0.08 |
| Myofibroblast |  | -0.07 |  |  | -0.25 |  |
| Epithelial Cell |  |  | -0.09 | -0.07 |  |  |
|  | *PGE2 Synthesis* | *Epithelial Cell TGF-β1 Binding Rate* | *Epithelial Cell Sensitivity to PGE2* |  |  |  |
| Fibroblast |  | -0.46 | -0.10 |  |  |  |
| Myofibroblast | -0.07 | -0.12 |  |  |  |  |
| Epithelial Cell |  |  |  |  |  |  |

**Table S5C: Significant PRCC Values for Cell Number at Day 3 (p<0.01)**

|  | *TGF-β1 Activation* | *TGF-β1 Receptor Dissociation* | *TGF-β1 Receptor Internalization Rate* | *TGF-β1 Receptor Recycling Rate* | *TGF-β1 Synthesis* | *PGE2 Binding Rate* |
| --- | --- | --- | --- | --- | --- | --- |
| Fibroblast |  |  |  |  |  | -0.16 |
| Myofibroblast | 0.09 |  |  | 0.07 | 0.51 |  |
| Epithelial Cell | -0.58 | -0.09 | -0.08 |  | -0.85 | 0.11 |
|  | *PGE2 Binding Rate* | *Fibroblast Sensitivity to PGE2* | *Fibroblast Insensitivity to TGF-β1* | *TGF-β1 Proliferation Threshold* | *PGE2 Proliferation Maximum* | *αSMA Synthesis Efficiency* |
| Fibroblast | -0.16 | 0.07 | 0.13 | -0.11 | 0.18 |  |
| Myofibroblast |  |  | -0.13 |  | -0.08 |  |
| Epithelial Cell | 0.11 |  | -0.16 |  | -0.08 | -0.07 |
|  | *αSMA Differentiation Maximum* | *TGF-β1 to PGE2 Differentiation Threshold* | *Myofibroblast TGF-β1 Binding Rate* | *PGE2 Synthesis* | *Epithelial Cell TGF-β1 Binding Rate* |  |
| Fibroblast | 0.09 | 0.17 |  | -0.43 |  |  |
| Myofibroblast |  | -0.26 | -0.08 | -0.13 | -0.09 |  |
| Epithelial Cell | -0.07 |  |  |  |  |  |

**Table S5D: Significant PRCC Values for Cell Number at Day 4 (p<0.01)**

|  | *TGF-β1 Activation* | *TGF-β1 Receptor Dissociation* | *TGF-β1 Receptor Internalization Rate* | *TGF-β1 Synthesis* | *PGE2 Binding Rate* | *Fibroblast Sensitivity to PGE2* |
| --- | --- | --- | --- | --- | --- | --- |
| Fibroblast |  |  |  |  | -0.16 | 0.08 |
| Myofibroblast | 0.10 |  |  | 0.52 |  |  |
| Epithelial Cell | -0.55 | -0.07 | -0.07 | -0.84 | 0.12 |  |
|  | *Fibroblast Insensitivity to TGF-β1* | *TGF-β1 Proliferation Threshold* | *PGE2 Proliferation Maximum* | *αSMA Synthesis Efficiency* | *αSMA Differentiation Maximum* | *TGF-β1 to PGE2 Differentiation Threshold* |
| Fibroblast | 0.14 | -0.09 | 0.17 |  | 0.08 | 0.19 |
| Myofibroblast | -0.14 |  | -0.07 |  |  | -0.26 |
| Epithelial Cell | -0.17 |  |  | -0.07 | -0.07 |  |
|  | *Myofibroblast TGF-β1 Binding Rate* | *PGE2 Synthesis* | *Epithelial Cell TGF-β1 Binding Rate* | *Epithelial Cell Sensitivity to PGE2* |  |  |
| Fibroblast |  | -0.42 |  | 0.07 |  |  |
| Myofibroblast | -0.07 | -0.12 | -0.09 |  |  |  |
| Epithelial Cell |  |  |  |  |  |  |

**Table S5E: Significant PRCC Values for Cell Number at Day 5 (p<0.01)**

|  | *TGF-β1 Activation* | *TGF-β1 Receptor Dissociation* | *TGF-β1 Synthesis* | *PGE2 Binding Rate* | *Fibroblast Insensitivity to TGF-β1* | *TGF-β1 Proliferation Threshold* |
| --- | --- | --- | --- | --- | --- | --- |
| Fibroblast |  |  |  | -0.16 | 0.13 | -0.11 |
| Myofibroblast | 0.11 |  | 0.53 |  | -0.13 |  |
| Epithelial Cell | -0.52 | -0.088 | -0.82 | 0.13 | -0.16 |  |
|  | *PGE2 Proliferation Maximum* | *αSMA Differentiation Maximum* | *TGF-β1 to PGE2 Differentiation Threshold* | *PGE2 Synthesis* | *Epithelial Cell TGF-β1 Binding Rate* | *Epithelial Cell Sensitivity to PGE2* |
| Fibroblast | 0.18 | 0.085 | 0.19 | -0.41 |  | 0.09 |
| Myofibroblast |  |  | -0.26 | -0.12 | -0.10 |  |
| Epithelial Cell |  |  |  |  |  |  |

**Table S5F: Significant PRCC Values for Cell Number at Day 6 (p<0.01)**

|  | *TGF-β1 Activation* | *TGF-β1 Receptor Dissociation* | *TGF-β1 Synthesis* | *PGE2 Binding Rate* | *Fibroblast Insensitivity to TGF-β1* | *TGF-β1 Proliferation Threshold* |
| --- | --- | --- | --- | --- | --- | --- |
| Fibroblast |  |  | -0.08 | -0.17 | 0.13 | -0.11 |
| Myofibroblast | 0.11 |  | 0.54 |  | -0.13 |  |
| Epithelial Cell | -0.49 | -0.08 | -0.81 | 0.13 | -0.16 |  |
|  | *PGE2 Proliferation Maximum* | *αSMA Differentiation Maximum* | *TGF-β1 to PGE2 Differentiation Threshold* | *PGE2 Synthesis* | *Epithelial Cell TGF-β1 Binding Rate* | *Epithelial Cell Sensitivity to PGE2* |
| Fibroblast | 0.16 | 0.08 | 0.20 | -0.41 |  | 0.08317 |
| Myofibroblast |  |  | -0.26 | -0.13 | -0.09 |  |
| Epithelial Cell |  | -0.08 |  |  |  |  |

**Table S5G: Significant PRCC Values for Cell Number at Day 7 (p<0.01)**

|  | *TGF-β1 Activation* | *TGF-β1 Receptor Dissociation* | *TGF-β1 Synthesis* | *PGE2 Binding Rate* | *Fibroblast Sensitivity to PGE2* | *Fibroblast Insensitivity to TGF-β1* |
| --- | --- | --- | --- | --- | --- | --- |
| Fibroblast |  |  | -0.11 | -0.17 | -0.12 | 0.12 |
| Myofibroblast | 0.10 |  | 0.54 |  |  | -0.13 |
| Epithelial Cell | -0.47 | -0.10 | -0.79 | 0.11 |  | -0.14 |
|  | *TGF-β1 Proliferation Threshold* | *PGE2 Proliferation Maximum* | *Probability of Fibroblast Movement* | *αSMA Differentiation Maximum* | *TGF-β1 to PGE2 Differentiation Threshold* | *PGE2 Synthesis* |
| Fibroblast | -0.11 | 0.14 | -0.08 | 0.07 | 0.19 | -0.40 |
| Myofibroblast |  |  |  |  | -0.26 | -0.14 |
| Epithelial Cell |  |  |  | -0.09 |  |  |
|  | *Epithelial Cell TGF-β1 Binding Rate* | *Epithelial Cell Sensitivity to PGE2* |  |  |  |  |
| Fibroblast |  | 0.07 |  |  |  |  |
| Myofibroblast | -0.09 |  |  |  |  |  |
| Epithelial Cell |  |  |  |  |  |  |

Supplemental Figures
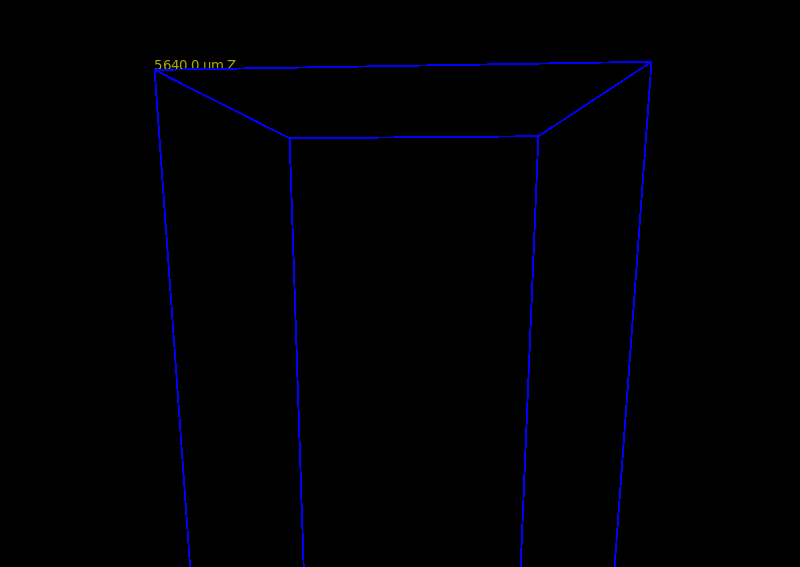


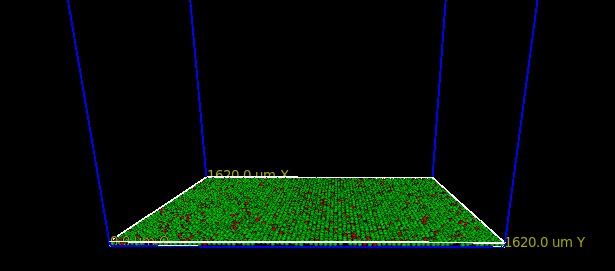


**Supplemental Figure 1:** Snapshot of the three-dimensional agent based model environment representing a virtual dish for the co-culture. Green spheres indicate epithelial cells while red spheres indicate fibroblasts. The blue lines indicated the edges of the boundaries of the ABM where media is present. Representative sections of top and bottom of the simulation environment are shown. The full simulation environment include 282 layers representing 6.17mm.The white lines indicate the edges of the boundaries of the cellular layer within the model (plastic bottom of well). Time lapse simulations of the model in 2D can be found at <http://malthus.micro.med.umich.edu/lab/movies/Co-culture/>.


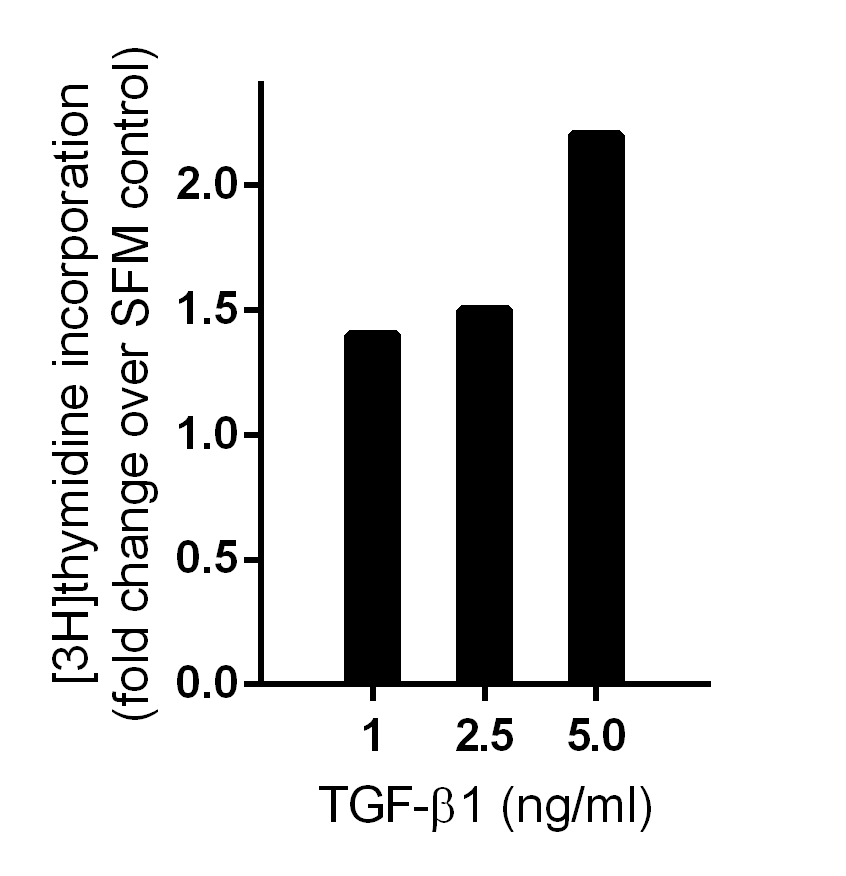


**Supplemental Figure 2**: Data reproduced from Hetzel et al. 2005 showing TGF-β1 induce fibroblast proliferation ([10](#_ENREF_10)). Normal human lung fibroblasts were cultured in either serum free media (SFM), SFM + 1.0ng/ml TGF-β1, SFM + 2.5ng/ml TGF-β1, or SFM + 5.0ng/ml TGF-β1.

**Bibliography**

1. Warsinske HC, Ashley SL, Linderman JJ, Moore BB, Kirschner DE. Identifying Mechanisms of Homeostatic Signaling in Fibroblast Differentiation. Bull Math Biol. 2015 Sep 18.

2. Lama V, Moore BB, Christensen P, Toews GB, Peters-Golden M. Prostaglandin E2 synthesis and suppression of fibroblast proliferation by alveolar epithelial cells is cyclooxygenase-2-dependent. Am J Respir Cell Mol Biol. 2002 Dec;27(6):752-8.

3. Hagimoto N, Kuwano K, Inoshima I, Yoshimi M, Nakamura N, Fujita M, et al. TGF-beta 1 as an enhancer of Fas-mediated apoptosis of lung epithelial cells. J Immunol. 2002 Jun 15;168(12):6470-8.

4. Shapiro BL, Feigal RJ, Laible NJ, Biros MH, Warwick WJ. Doubling time alpha-aminoisobutyrate transport and calcium exchange in cultured fibroblasts from cystic fibrosis and control subjects. Clin Chim Acta. 1978 Jan 2;82(1-2):125-31.

5. Wieser RJ, Heck R, Oesch F. Involvement of plasma membrane glycoproteins in the contact-dependent inhibition of growth of human fibroblasts. Exp Cell Res. 1985 Jun;158(2):493-9.

6. Wieser RJ, Janik-Schmitt B, Renauer D, Schafer A, Heck R, Oesch F. Contact-dependent inhibition of growth of normal diploid human fibroblasts by plasma membrane glycoproteins. Biochimie. 1988 Nov;70(11):1661-71.

7. Duhamel P, Vetterli M. Fast Fourier-Transforms - a Tutorial Review and a State-of-the-Art. Signal Process. 1990 Apr;19(4):259-99.

8. Cilfone NA, Kirschner DE, Linderman JJ. Strategies for efficient numerical implementation of hybrid multi-scale agent-based models to describe biological systems. Cell Mol Bioeng. 2015 Mar;8(1):119-36.

9. Strutz F, Zeisberg M, Renziehausen A, Raschke B, Becker V, van Kooten C, et al. TGF-beta 1 induces proliferation in human renal fibroblasts via induction of basic fibroblast growth factor (FGF-2). Kidney Int. 2001 Feb;59(2):579-92.

10. Hetzel M, Bachem M, Anders D, Trischler G, Faehling M. Different effects of growth factors on proliferation and matrix production of normal and fibrotic human lung fibroblasts. Lung. 2005 Jul-Aug;183(4):225-37.

11. Vilar JM, Jansen R, Sander C. Signal processing in the TGF-beta superfamily ligand-receptor network. PLoS Comput Biol. 2006 Jan;2(1):e3.

12. Hayley C. Warsinske SLA, Jennifer J. Linderman, Bethany B. Moore, Denise E. Kirschner. Identifying Mechanisms of Homeostatic Signaling in Fibroblast Differentiation. Bulletin of Mathematical Biology. 2015 2015/9/3(11538):27.

13. Wakefield LM, Winokur TS, Hollands RS, Christopherson K, Levinson AD, Sporn MB. Recombinant latent transforming growth factor beta 1 has a longer plasma half-life in rats than active transforming growth factor beta 1, and a different tissue distribution. J Clin Invest. 1990 Dec;86(6):1976-84.

14. Jin J, Zhang T. Effects of glucose restriction on replicative senescence of human diploid fibroblasts IMR-90. Cell Physiol Biochem. 2013;31(4-5):718-27.
